# Insights into Protein–DNA Interactions through Structure Network Analysis

**DOI:** 10.1371/journal.pcbi.1000170

**Published:** 2008-09-05

**Authors:** R. Sathyapriya, M. S. Vijayabaskar, Saraswathi Vishveshwara

**Affiliations:** Molecular Biophysics Unit, Indian Institute of Science, Bangalore, India; National Cancer Institute, United States of America and Tel Aviv University, Israel

## Abstract

Protein–DNA interactions are crucial for many cellular processes. Now with the increased availability of structures of protein–DNA complexes, gaining deeper insights into the nature of protein–DNA interactions has become possible. Earlier, investigations have characterized the interface properties by considering pairwise interactions. However, the information communicated along the interfaces is rarely a pairwise phenomenon, and we feel that a global picture can be obtained by considering a protein–DNA complex as a network of noncovalently interacting systems. Furthermore, most of the earlier investigations have been carried out from the protein point of view (protein-centric), and the present network approach aims to combine both the protein-centric and the DNA-centric points of view. Part of the study involves the development of methodology to investigate protein–DNA graphs/networks with the development of key parameters. A network representation provides a holistic view of the interacting surface and has been reported here for the first time. The second part of the study involves the analyses of these graphs in terms of clusters of interacting residues and the identification of highly connected residues (hubs) along the protein–DNA interface. A predominance of deoxyribose–amino acid clusters in β-sheet proteins, distinction of the interface clusters in helix–turn–helix, and the zipper-type proteins would not have been possible by conventional pairwise interaction analysis. Additionally, we propose a potential classification scheme for a set of protein–DNA complexes on the basis of the protein–DNA interface clusters. This provides a general idea of how the proteins interact with the different components of DNA in different complexes. Thus, we believe that the present graph-based method provides a deeper insight into the analysis of the protein–DNA recognition mechanisms by throwing more light on the nature and the specificity of these interactions.

## Author Summary

The interaction of proteins with DNA is crucial for several cellular processes. Some insights into the mode of interaction can be obtained from the analysis of the complexed structures. Conventional analyses are based on the identification of pairwise interactions. However, a collective representation of the network of interactions and the analyses of such networks provide valuable information, which is not easy to obtain from pairwise analyses. Although the protein structure networks have been described in the literature, this is the first time that a network representation of protein–DNA is described. Construction and analysis of such networks have given valuable information on protein–DNA interactions in terms of network parameters, such as clusters of interacting residues and hubs, which are highly connected residues. Furthermore, the results also represent both the protein- and the DNA-centric viewpoints, because the analysis is carried out on combined networks. The methodology developed here can lead to predictions, such as important residues responsible for stabilizing protein–DNA interactions, and will be of interest to experimentalists.

## Introduction

A network of interactions among the macromolecules drives the cell. The protein–DNA interactions orchestrate the high fidelity processes like DNA recombination, DNA replication, and transcription. With the increasing number of high-resolution structures of macromolecular complexes, it is now possible to obtain insights into the atomic details of interactions governing their structural and functional integrity. In the present study, we focus on protein–DNA interactions, which can either be specific or non-specific depending on the functional requirement. Insights into the mechanism of protein–DNA binding and recognition have come from extensive analysis of protein–DNA interfaces [1]–[14]. Some of these investigations have been carried out at the level of pairwise interactions between the atoms/residues of the interacting partners. However, the information communicated along the interfaces is rarely a pairwise phenomenon. New insights can be gained by investigating the interactions holistically, extending beyond the pairwise analysis of atomic/residue interactions. This can be achieved through the use of efficient methods, which capture the topological features from the structures of these complexes. The concept of representing protein structures as graphs exists in the literature [15]–[21]. In these studies the amino acids in proteins are considered as nodes and the interaction between these nodes have been considered as edges for constructing different types of graphs. These protein structure graphs (PSG) have been successfully used in the analysis of protein structure, stability and function [22],[23]. PSGs have also been analyzed in protein–DNA complexes to identify significant interactions as clusters of interacting amino acids at the protein–DNA interfaces [4]. However in such studies, the interacting nucleotides of the DNA were not considered as part of the graphs, since the parameters required for representing DNA as graphs were not available at that time.

As a conceptual turning point, it has been pointed out that most of the information on protein–DNA complexes have been obtained from a “protein-centric” view and new insights are likely to emerge if protein–DNA complexes are investigated from a “protein–DNA-centric” viewpoint [24]. Recently, protein–DNA complexes have been classified on the basis of structural descriptors that highlights the significance of the protein induced distortions of the DNA [3]. In the current article, the interactions between the protein and the DNA in protein–DNA complexes have been evaluated in a collective fashion by considering the complexes as bipartite graphs. We have developed the parameters to evaluate the strength of interaction between the amino acids and the nucleotides, based on the atomic contacts. Such a combined graph based analysis of protein–DNA interactions has been presented for the first time in this study.

The protein–DNA graph is of special interest, since we are dealing with two different types of biopolymers with unique structural and chemical properties. In the case of proteins, the two amino acids are linked by a rigid peptide bond and each amino acid could be unambiguously considered as a node in the protein graph. However, in the case of DNA, the linkage between two nucleotides is through a flexible phosphodiester bond and the nodes can be defined at various levels. For example, a nucleotide as a whole or its individual chemical components such as the phosphate group, deoxyribose and the bases (A, T, G, and C) can be represented as nodes. Such a different representation of nodes has distinct advantages of their own, in interpreting the nature of interaction between the protein and DNA [13], as we will show in this study. An important component of the analysis is to quantify the strength of interaction (on the basis of the number of atomic contacts) between these chemically different molecules to capture the essence of the intermolecular interactions at the protein–DNA interface.

The present graph based analysis of protein–DNA complexes focuses on the following points. Primarily, the interface interactions of protein–DNA complexes have been investigated at a network level. This is achieved by constructing protein–DNA graphs (PDGs) on the basis of the strength of interaction between the nodes and also by performing extensive calibrations to choose the optimal strength of interactions to gain structural insights. Secondly, the clusters and hubs of such interacting amino acids and the nucleotides at the interfaces have been analyzed in a set of protein–DNA complexes.

Significant results that are inaccessible by conventional pairwise analysis of the structure or by sequence analysis have been obtained from the present work. These include the identification of spatial networks of interacting residues that are sequentially far apart, the evaluation of a scale of interaction strength along which we can compare and analyze the interaction networks of protein–DNA complexes and the identification of groups of optimally interacting residues which stabilize the structural architecture. Furthermore, we have been able to revisit the classification scheme of DNA binding proteins. Our classification schema, which is based on the concepts of graphs/network, interaction strength, and the type of interaction, is distinct from the classification schemes proposed earlier with a protein-centric point of view. We have compared our results with the other classification schemes [3],[25].

## Results/Discussion

### Clusters as a Function of MEC at the Protein–DNA Interface

**Figure 1:**
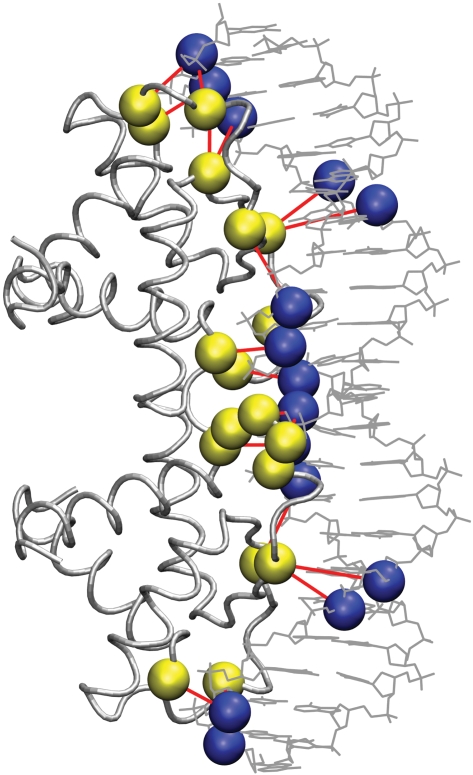
A bipartite graph representation of the amino acid–nucleotide interactions in a protein–DNA complex. In the bipartite graph representation of the protein–DNA complex, the amino acids and the nucleotides of the DNA form the two node sets. Shown in yellow are amino acid nodes, and blue, the nucleotides. The edges (between the nodes from one set to the other) are shown in red. These edges are defined based on a specific MEC. The MEC quantifies the minimum number of atomic contacts expected between an amino acid and a nucleotide to define an edge (Equation 1). The contacts are specifically evaluated between side chains of the amino acids and the phosphate, or the deoxyribose sugar or the base of nucleotides to form the P-p, P-S, and P-B clusters, respectively.

A protein–DNA graph (PDG) is a bipartite graph constructed to represent the interaction between the amino acids of the protein and the nucleotides of the DNA in a protein–DNA complex. A bipartite graph deals with two different node sets and edges are defined across the two node sets. A contact in the bipartite PDG is defined when a side chain of an amino acid interacts with the nucleotide. The interactions of the amino acid with the nucleotide can be considered at different levels: with the phosphate (p), deoxyribose sugar (S) or base (B) components individually, or with the nucleotide as a complete entity. The edges are defined upon quantification of the interaction between the amino acids and the nucleotides with the “Interaction Strength,” *I_ij_* (It is to be noted that the interaction strength mentioned here is based on the number of atom-atom contacts and in a way reflects only the local packing density.) The details of the construction of PDG are presented in the Materials and Methods section. A bipartite PDG representation of a protein–DNA complex is illustrated in Figure 1.

The nodes in a PDG are connected if the *I_ij_* evaluated between the nodes is greater than or equal to a user-defined *I_ij_*. The user-defined *I_ij_* is termed as Minimal Effective Connection (MEC). We have constructed PDGs from protein–DNA complexes using a range of MEC from 0% to 15%. A graph generated with a high MEC is sparse with strongly connected nodes, and the graph generated with a low MEC is dense with weakly connected nodes (Figure S1). Hence, the choice of the window of MEC for the analysis of the graphs becomes important, which ensures that we neither include insignificant (weak) interactions nor miss significant (strong) ones. As we have partitioned the nucleotide of the DNA into its phosphate, deoxyribose and base components, an optimal range of MEC (OMEC) is chosen for constructing protein-phosphate (P-p), protein-deoxyribose (P-S) and protein-base (P-B) graphs. The OMEC is selected to balance a trade-off between the strength of interaction and the cluster size. Thus, the MEC is further classified into weak (WMEC), optimal (OMEC) and strong (SMEC) on the basis of *I_ij_* and the ranges of MEC values are listed in Table 1. All further discussions are based on the analyses with the OMEC ranges, unless otherwise specified. We obtain P-p, P-S, and P-B clusters and hubs from the adjacency matrices of PDGs (see Materials and Methods section). The detailed analysis of protein–DNA complexes through these parameters is presented in the following section.

**Table 1:** Range of minimal effective connection (MEC).

Graph	Weak (WMEC)	Optimal (OMEC)	Strong (SMEC)
Protein-Phosphate (P-p)	Up to 3%	3% to 5%	Above 5%
Protein-Deoxyribose (P-S)	Up to 4%	4% to 8%	Above 8%
Protein-Base (P-B)	Up to 3%	3% to 5%	Above 5%

The rationale for selecting these ranges is mentioned in Supporting Information.

### Amino Acid Propensities in PDGs

**Figure 2:**
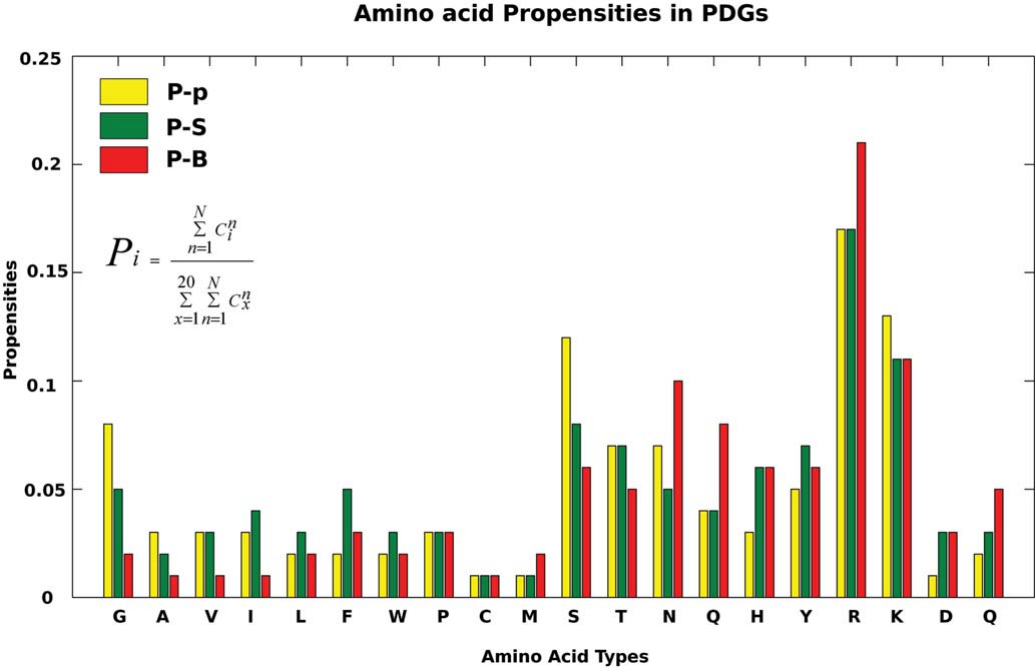
Amino acid propensities in PDGs. Amino acid propensities in the different component graphs are calculated in the OMEC range. The propensity for a particular amino acid “*i*” (*P_i_*) is calculated as 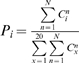 where 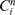 is the number of occurrences of the amino acid “*i*” in a PDG of protein–DNA complex “*n*” and *N* is the total number of structures in the dataset. The figure shows the propensities of amino acids in all the component graphs across the whole dataset.

The propensities of amino acids to form P-p, P-S, and P-B graphs were calculated from DS1 (see Materials and Methods section). The results are presented in Figure 2. In general, we observe a higher propensity of basic residues (Arg and Lys) to occur in PDGs. Arg is more preferred in P-B graphs whereas Lys is more preferred in P-p graphs. All polar amino acids occur significantly in all the three component clusters, however the preferences vary. For instance, Ser, the smallest polar amino acid has a higher propensity to occur in P-p graphs, and Asn/Gln, which contain the planar conjugated amide group has higher propensity of occurrence in P-B graphs. The interactions of Val, Ile, Leu, Phe, Trp are higher in the P-S graphs indicating that the deoxyribose is involved in hydrophobic and van der Waals interactions. The acidic residues (Asp and Glu) are not excluded from the interface interactions, in spite of the net negative charge of DNA. However, the occurrence of these amino acids near the phosphate backbone (P-p graphs) is minimal and significant near the bases (P-B graphs). Also, Glu interacts with bases more frequently than Asp. Thus, the analysis confirms some of the expected trends of interaction between amino acids and DNA, such as the dominance of basic and polar residues, and lesser preference of hydrophobic and acidic amino acids in PDGs. Additionally, the preference of interactions with individual chemical components such as the phosphate backbone, deoxyribose sugar and bases have been elucidated in detail. Such a level of analysis is useful in understanding the subtleties involved in protein–DNA interactions, and for interpreting the nature of component graphs as will be discussed later in the context of classification of protein–DNA complexes.

### Cluster Profiles of Different Groups of Protein–DNA Complexes

The protein–DNA complexes have been classified into different groups based on the structural similarity of the proteins bound to the DNA. Luscombe et al have provided a comprehensive classification of the protein–DNA complexes based on the secondary structural motifs of proteins interacting with the DNA [25]. The classification results in eight groups of complexes: β-sheet group, β-hairpin group, helix turn helix (HTH), zipper type (ZT), zinc coordinating group, other α-helices, enzymes and others [25]. We have cured it further to remove structures with single-stranded DNA (see Table S1 for details regarding the members of the dataset). We have generated PDGs for all groups of protein–DNA complexes. The PDGs are further analyzed to investigate the properties such as the preference of proteins to interact with the DNA, the components of the DNA to which the protein binds, the dominance of a particular type of cluster (P-p, P-S, or P-B). We have also tried to find a generic pattern (if any) of clusters that could be identified amongst the groups of protein–DNA complexes. The important results for each group are discussed in the following sections.

#### β-Strand groups

β-Sheet group and the β-hairpin group are the two well defined groups that use β-strands as the major secondary structure to interact with the DNA. Although the presence of the β-strand is a common feature in these groups, the modes of interaction are distinctly different from one another, as will be discussed below.

**Figure 3:**
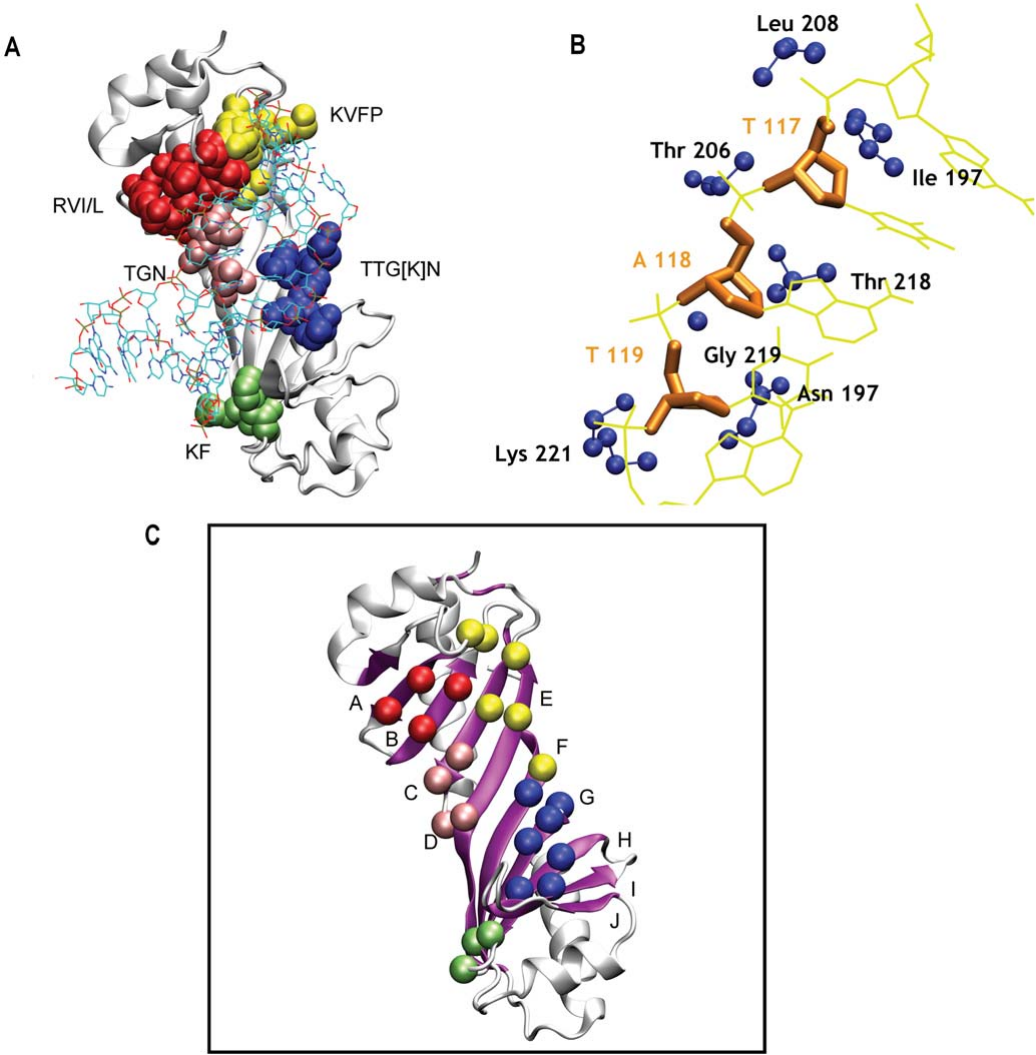
Protein-deoxyribose (P-S) clusters in β-sheet group. (a) Different P-S clusters in the TATA binding protein (1tgh) (at MEC 7%) are shown in different colors with their amino acid composition. (b) A P-S cluster is shown in detail to reveal the tight interactions between the amino acids (blue) and the deoxyribose (orange) of the nucleotides. (c) Only the Cα of the amino acids of the P-S clusters is highlighted in the sheets named from A to J.

*β-Sheet.* β-Sheet group uses an anti-parallel β-sheet to interact with the minor groove of DNA. This group comprises the TATA box binding family, which is involved in promoter binding and transcription initiation of the associated gene. Given the tendency of the β-sheets to twist, they provide a saddle like scaffold on which the minor groove of the DNA is well seated [26]–[28]. We find an overwhelming dominance of P-S clusters compared to P-p or P-B clusters in the members of this group. These P-S clusters (consisting of amino acids from the protein and the deoxyribose units of DNA) are present in the β-sheet region and interact with the DNA as shown in Figure 3a and 3b. The amino acid composition of the clusters in this group of DBPs is given in Table 2. We observe that the P-S clusters, which appear in the minor groove, are located in similar positions and their amino acid compositions are very similar among the members of this group. For instance, most of the P-S clusters: TGN, RVIL, KVFP, TTGN, and KF (Figure 3a) are observed as a common pattern from this group. The residues in these clusters come from the sheet whose strands are labeled from A to J (Figure 3c). TGN is a polar cluster present near the center of the β-sheet with Asn at the center of the strand E while Thr and Gly come from the strand D. The RVIL cluster is a hydrophobic cluster which is present at the end of the β-sheet (strands B, C). The residues Arg and Ile come from the middle of the strand B while Val and Leu are being donated by the neighboring strand C. The KVFP cluster is situated near RVIL cluster with Lys and Val coming from the strand D, whereas Phe and Pro come from the loops (Figure 3c). The cluster TTGN[K] is similar to the combined clusters of TGN and RVIL and located in a position which is almost symmetrical to these clusters. The cluster KF occupies a position which is symmetrical to the cluster KVFP. Here we should note that a Pro residue also exists near the KF cluster, equivalent to the symmetrical KVFP cluster, but does not form a part of the interface cluster. This is due to the reason that the Pro near KF cluster is present away from the protein DNA interface, making no contacts with the nucleotides.

**Table 2:** Commonly occurring P-S clusters (at MEC = 5%) in β-sheet proteins.

PDBS	Cluster number and composition								
	1	2	3	4	5	6	7	8	9
**1ais**	TGN	KVFP	RVI						
**1cdw**	TGKN	KVFP-SQ	RVIL	ILTTGKN	KSF	FL			
**1d3u**	TGKNA	KVFP	RVILNGQS				VFP		
**1c9b**	TGN	KVFP	RVL	RLTTGN	KVSF				
**1tgh**	TGNILV	KVFP		ILTTGKN	KVF	FL		FR	
**1vol**	TGN	KVFP	RVILF	TTGKN	KF				RLI
**1ytf**	TGN	KVFP	RVI	ILTTGKN	KF				

Note that the nucleotides involved in the clusters are not reported. Complete information including the nucleotides in these clusters, residue number, and chain is given in Table S2.

**Figure 4:**
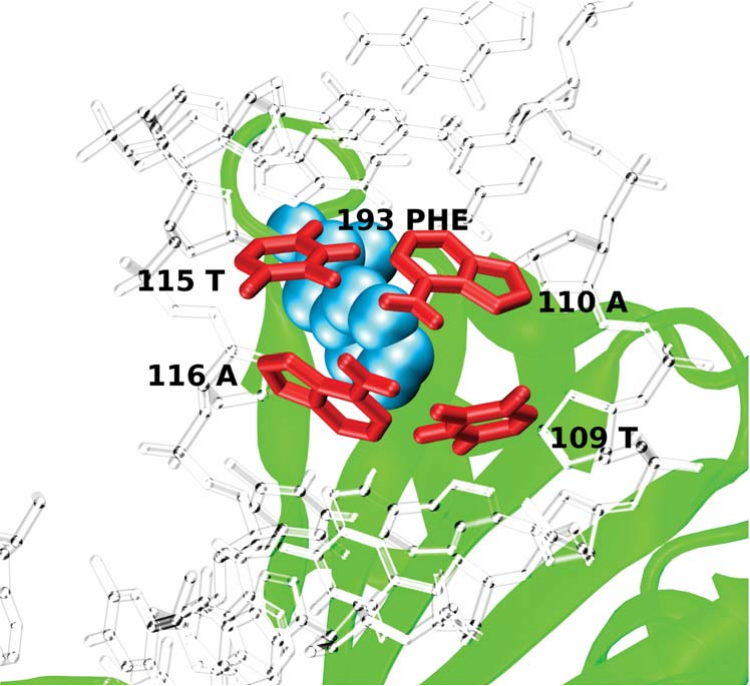
A protein-base (P-B) hub in TATA box binding protein (TBP/DNA). An amino acid interacts with four different bases to form a P-B hub. In the TATA box binding protein (1tgh) the position of a P-B hub Phe193 (at MEC 3%) is shown. This hub (blue) interacts mainly with bases from four different nucleotides (red) T115, A116, T109, and A110.

Hubs have been defined as amino acid residues which are connected to four or more nucleotides or vice versa. The members of this group contain a few hubs (Table 3 and Table S3). Most characteristic feature of this group is the presence of Phe (P-B) hub in all the members of the group. It is interesting to note that this structurally important residue identified as a hub is observed at the DNA bending region (Figure 4) and could be correlated to the deformation of the DNA. Thus, the protein-induced DNA deformation, which was observed earlier [28], is elegantly captured here by this network property.

**Table 3:** Hubs identified in the component graphs of DNA-binding proteins belonging to β-hairpin, β-sheet, and zipper-type groups.

PDBS	P-p	P-S	P-B
**β-Hairpin**			
Integration host factor	C-42C C-41A C-31G C-29A C20T D24A E36C E46C	B46ARG	
**β-Sheet**			
Human TATA box binding protein		B7T B8A C107T C108T	A284PHE
**Zipper type**			
GCN4	A6A A7T B28A		C235ASN D235ASN

The nodes in the clusters are given as chain id-residue id residue name. (MEC_(P-p)_ = 3%, MEC_(P-S)_ = 4%, MEC_(P-B)_ = 3%). Only a representative from each group is given. Other members of the group are given in Table S3.

The analysis of the patterns of clusters from PDG of the β-sheet group has thus revealed striking features like the presence of consistent P-S clusters across all the members of this group. Further, the presence of a Phe hub in the graph is also linked to the deformation of the DNA. These consistent patterns that emerge through our method of bipartite graph representation are not only qualitatively similar in terms of the residue composition, but also possess a similar connectivity between the nodes of the graph as evaluated with the MEC.

**Figure 5:**
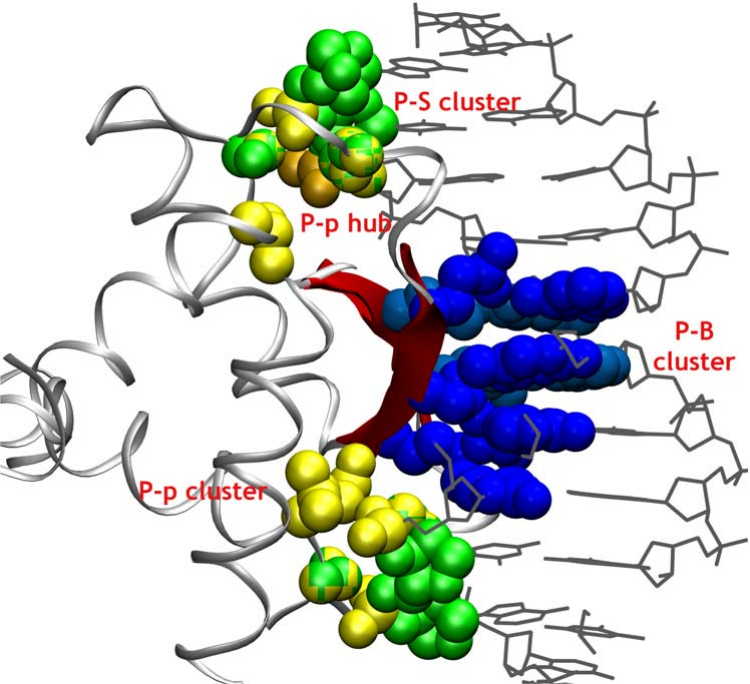
P-p, P-S, and P-B clusters in the β-hairpin group protein Arc (1bdt). In the Arc protein (1bdt), the P-p (yellow), P-S (green), and P-B (blue) clusters are shown. The β-hairpin that is interacting with the DNA is highlighted in red. A cluster of charged residues (Arg, Asn, and Gln) from the β-hairpin makes contact with successive bases of the DNA. These P-B clusters are flanked by the P-S and P-p clusters arising from other secondary structures (helices and loops) around the β-ribbon. A P-p hub (orange) in which the phosphate group of Ade4 interacts with Ser32, Val33 and Phe10 is also highlighted.

*β-Hairpin.* The interaction motif of this group comprises two small β-strands and a loop (also known as the hairpin motif), which interacts with either the major or the minor groove of the DNA. Unlike the β-sheet groups, the members of this group have diverse structures, albeit a common interaction scheme at the interface. Also, this group contains all the three (P-p, P-S, P-B) clusters. The P-S clusters are significantly less compared to the β-sheet group. Most members of this group have a characteristic P-B cluster located in the β-strand region that interacts with the major groove of the DNA (Figure 5). It can further be seen that the P-S and the P-p clusters are located at regions surrounding the β-hairpin and not directly at the β-hairpin region.

Discrimination of the β-sheet group and the β-hairpin group is also brought out by hub analysis. We observe that P-p hubs are dominant in the β-hairpin group, in contrast to the P-S hubs observed in the β-sheet group (Table 3 and Table S3). A common feature of the P-p hubs of all the β-hairpin members is the absence of this hub in the main β-hairpin region. The positions of the P-p hubs vary with respect to the interacting β-hairpin in this group. For, e.g., Arc protein/DNA complex (1bdt) and Met Repressor/DNA Complex (1cma) has P-p hubs in the flanking region adjacent to the β-hairpin (Figure 5), whereas Integration Host Factor/DNA Complex (1ihf) has the hubs scattered around the β-hairpin interaction region. The P-p hubs in T Domain-DNA complex (1xbr) are located on either ends of the complexed DNA.

#### Helix groups

Groups like helix turn helix (HTH), zipper-type (ZT), zinc coordinating group (ZC), and other α-helix group (OAH) use helices as the major secondary structure to interact with the DNA. HTH and ZT groups have been classified based on their structural motifs, while ZC group is classified according to the presence of coordinated Zinc in the complex. The Other α-Helix group constitutes all the remaining proteins that employ α-helices to interact with DNA. We center our discussion mainly on the well-characterized HTH and ZT groups.

**Figure 6:**
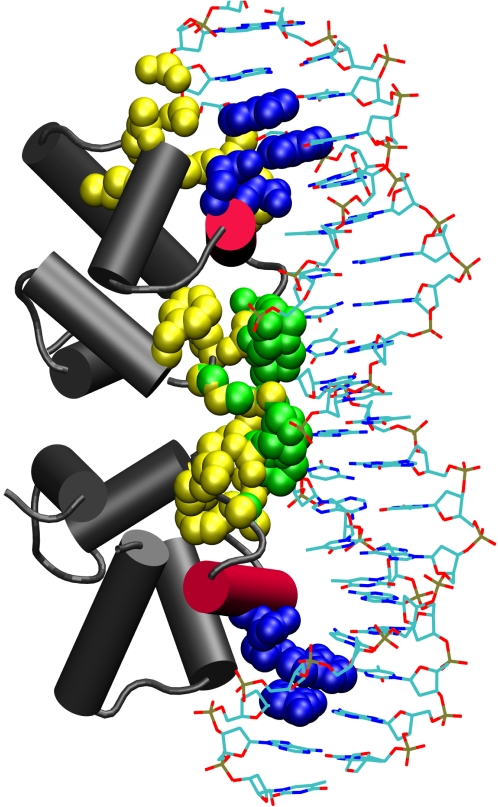
Clusters (P-p, P-S, and P-B) from the 434 repressor protein of the HTH group. The P-p (yellow), P-S (green), and P-B (blue) clusters are shown in the bacteriophage 434 repressor protein (1rpe) which belongs to the HTH group. The P-B clusters are present in the recognition helix that interacts with the major groove of the DNA and the P-p and the P-S clusters are present in the other interacting region that interacts with the minor groove.

*Helix turn helix.* A HTH motif consists of two helices, which are almost perpendicular to each other, linked by a turn or a loop. One of these helices, the recognition helix, binds to the major groove, interacting with the exposed bases and the other helix is known as the probe helix [25],[29]. Our cluster analysis has shown that the recognition helix almost always hosts a P-B cluster involving amino acid residues that interact with the exposed bases at the major groove (Figure 6). The other parts of the motif, like loops or turns or the probe helix are involved in forming P-S or P-p clusters. These clusters could provide a scaffold such that the P-B clusters bind efficiently at the major groove (Figure 6). While these features are common to almost all the members of this family, minor variations are seen in terms of the size and the number of these clusters (details can be obtained from Table S2).

**Figure 7:**
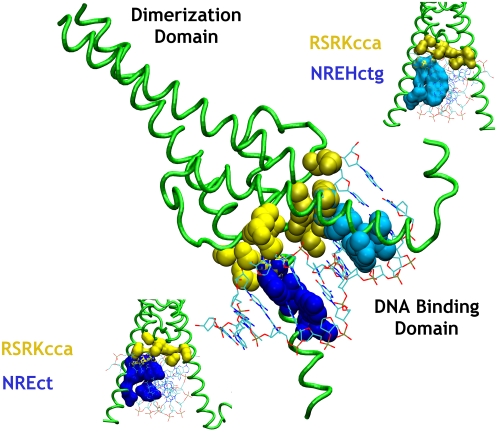
Interaction of the transcription factor Max (1hlo) of the zipper-type group (basic-helix-loop-helix-zipper) with DNA. The Max transcription factor, which belongs to the Helix-Loop-Helix Zipper family, has a dimerization domain and a DNA binding domain (center). The DNA binding domain shows the presence of symmetric P-p (yellow) and asymmetric P-B clusters (blue) (upper and lower). The cluster compositions are given (single letter code for nodes: upper case for amino acids and lower case for nucleotides). The cluster compositions for other proteins are given in Table S2. There is a marked absence of P-S clusters in the recognition of DNA by this zipper protein.

*Zipper-type.* Two helices are present in the zipper-type group (basic-zipper family and helix-loop-helix zipper family belong to this group). They consist of a dimerization domain and a DNA binding domain. The dimerization domain is a coiled coil helix with hydrophobic amino acids at the C terminal end. In the N terminal end, the helices diverge to bind to the opposite faces of the major grooves, looking like prongs holding the DNA double helix (Figure 7). Significant P-p and P-B clusters have been identified in this group (Figure 7 and Table S2). Especially P-p clustering is extensive and consistent in all the members of the group. P-S clusters and hubs are rarely found (Table 3). In contrast to the distinct recognition and the probe sites in HTH motif, the entire interaction of the protein with DNA is localized to a small region in the ZT group as depicted in Figure 7.

There have been studies of asymmetric base pair recognition by the zipper type proteins and the dimers binding DNA in a specific orientation [30],[31]. This asymmetry can be readily seen in the pattern of P-B clusters formed from both the monomers (Figure 7). Each monomer interacts independently at the major groove of the DNA and different (asymmetric) P-B clusters are formed by the monomers. For e.g. in GCN4 bound to DNA containing the pseudo-palindromic AP1 site, there are two additional nodes (Ser242 and an additional A35) in one of the P-B clusters. We noticed that such an asymmetry in P-B clusters became more pronounced with increasing MEC (data not shown). This phenomenon shows that one of the monomer binds/packs more strongly to the bases of the nucleotides than the other. Sequentially highly conserved residue Asn235 [32] has been found to be both an important node in the P-B cluster and also a P-B hub in GCN4. Also, the degree of asymmetric behavior can be quantified from the difference in the number of nodes involved in the P-B clusters from the monomers. Furthermore, we observe that though the P-B clusters are asymmetric the P-p clusters are symmetric (Figure 7). Such a feature seen for example, in Max/DNA complex indicates that the base specific interactions of the proteins may be asymmetric, whereas the non-specific electrostatic interactions of the protein with the DNA can be symmetric. Thus, the cluster patterns quantitatively differentiate the asymmetric binding of the chains to the major grooves of the DNA.

*Other α-helices.* All the other proteins that use helices as their major secondary structure to interact with the DNA are classified under the other α-helix group. This group has diverse members describing the possible ways in which a helical motif can interact both specifically or non-specifically with DNA, presenting interesting cases to study in detail. The size and the oligomerisation state of the members of this group vary considerably. The diversity of protein structures and their interactions with DNA are also reflected in the interface clusters (Table S2). For example, the bigger members of this group like nucleosome core particle (1aoi) (histone–DNA complex), EBNA1 (Epstein Barr virus origin binding protein)–DNA complex (1b3t), Yeast MATα2/MCM1(MADS Box Transcription Factor)/DNA ternary complex (1mnm) have extensive P-p clusters, while the smaller members like high-mobility protein (1ckt), HMG-D/DNA complex (1qrv), skn-1 binding domain–DNA complex (1skn) do not exhibit extensive P-p clustering. P-S clusters can be extensive as in 1mnm, 1aoi or weak in the case of 1skn. P-B clusters are present in the vicinity of the kinked region of DNA as in the case of 1ckt and 1qrv. The P-B clusters are also present at the protein-protein interfaces in oligomeric proteins, which make contact with DNA.

A detailed investigation of the nucleosome core particle (1aoi) is presented below.

*Nucleosome core particle.* In eukaryotic genomes, the packing and assembly of DNA into higher order structures (chromatins) is originated by the initial binding of DNA to histones to form nucleoprotein complexes. They play an important role as a packaging element that determines the accessibility of the DNA to other factors and enzymes of the DNA replication, transcription and repair machineries [33]–[36]. The nature and the organization of about 145–147 base pairs of DNA around the octameric histones has been unveiled by the X-ray structure of the nucleosome core particle by Luger et al (1aoi) [34]. Another structure of histone octamer complexed with DNA was solved (1s32) in the presence of a polyamide linker clamping the two gyres of DNA [37]. This clamp was shown to stabilize the histone-DNA complex [37]. We investigated this structure also, to understand the effect of clamping on the clusters. First, we ensured that the clamping indeed brought the two gyres of DNA closer. The turn involving the linker region has been compressed by 2.1 Å and the neighboring turns by 0.4 and 0.9 Å as evaluated by the nearest phosphate distances between the two gyres of DNA.

We have generated P-p, P-S, and P-B graphs for these two histone-DNA complexes (1aoi and 1s32). There is a clear domination of P-p and P-S clusters and a complete absence of P-B clusters. The lack of specific P-B clusters and the predominance of backbone mediated non-specific clusters agree with the fact that the histones interact non-specifically with the DNA through the electrostatic [38] and van der Waals interactions. This is a plausible explanation as to why a histone can reversibly bind and can unwind later without causing considerable structural distortion to the DNA.

**Figure 8:**
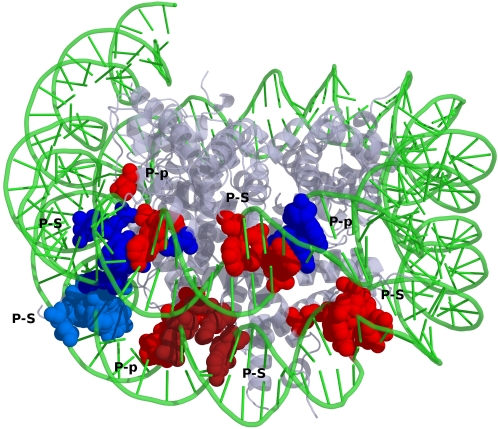
An overview of differences in the pattern of clusters observed due to the presence of linker in the nucleosome core particle. The superposition of the two structures is done using Align. (Only 1aoi is represented in the above figure for clarity.) The clusters that remain unperturbed in both the structures are given in red. The new clusters that are formed due to the conformational changes mediated by the presence of the linker are given in blue. Significant P-B clusters were not seen in both the structures. The composition of the clusters in the linker region is given in Table S4.

The cluster analysis of these two structures has shown that there are four additional P-p clusters in the clamped structure out of which one is around the linker region. There are five additional P-S clusters and two are present near the linker region (Figure 8). These observations suggest that the linker induced compression of the DNA has brought about an increased interaction of the DNA with the histone octamer. Interestingly, this compression has increased only the non-specific electrostatic and van der Waals interactions with the phosphate and sugar moieties. No significant change in P-B clusters suggests that the compression in the presence of the linker does not significantly affect the binding specificity of the histone.

### Classification of Protein–DNA Complexes

The importance of classifying the DNA binding proteins from the protein–DNA point of view, rather than the protein-centric view is being recognized for developing better protein–DNA recognition code [24]. Since our method considers the spatial relationships between the amino acids and the nucleotides at the interface, we have attempted to classify the DNA binding proteins based on these spatial relationships.

The interface P-p, P-S, and P-B clusters are examined in the protein–DNA complexes. The analysis of these clusters shows that the complexes can either have exclusive P-p, P-S, or P-B clusters or they can contain a mixture of these types of clusters. In cases where more than one type of cluster is observed, we define overlapping (amino acids sharing the same nucleotide) or non-overlapping (amino acids making contact with different nucleotides) clusters. Based on the types of clusters observed, the complexes are classified into seven groups. The complexes containing exclusive P-p, P-S, and P-B clusters are denoted as class 1, 2, and 3, respectively. Mixtures of (P-p+P-S), (P-S+P-B), and (P-p+P-B) clusters are denoted as class 4, 5, and 6, respectively. The complexes containing all the three component clusters (P-p, P-S, and P-B) are considered as class 7. A sub-classification based on the presence of overlapping or non-overlapping (P-p+P-S) or (P-S+P-B) or (P-p+P-B) components is made for cases 4 to 7. The details of the classification of the protein–DNA complexes are presented in Table 4. Among the protein–DNA complexes of the dataset only ten complexes exhibit exclusive P-p, P-S, or P-B clusters at the protein–DNA interface and thereby fall into distinct classes 1, 2, or 3 (Table 4). Majority of the other complexes however, seem to employ concerted interactions and interact with different chemical components of the nucleotide by forming two or more types of clusters (either overlapping or non-overlapping) at the interface (see Figure S2 for illustrative figures).

**Table 4:** Bipartite Graph based classification of DNA-binding proteins.

Class 1	Class 2	Class 3	Class 4		Class 5		Class 6		Class 7				
P-p clusters only	P-S clusters only	P-B clusters only	P-p and P-S clusters (no P-B clusters)		P-S and P-B clusters (no P-p clusters)		P-p and P-B clusters (no P-S clusters)		P-p, P-S, and P-B clusters are present				
			Overlapping clusters	Non-overlapping clusters	Overlapping clusters	Non-overlapping clusters	Overlapping clusters	Non-overlapping clusters	Overlapping P-p, P-B, and P-S clusters	Non-overlapping P-p, P-B, and P-S clusters			
										P-p and P-S clusters overlap but not P-B clusters	P-S and P-B clusters overlap but not P-p clusters	P-p and P-B clusters overlap but not P-S clusters	P-P, P-B and P-S clusters occur separately
**β-Hairpin**	**β-Hairpin**	**Zinc coordinating group**	**Enzymes**	**β-Hairpin**	**β-Hairpin**	**Other α-helices**	**Others**	**Helix turn helix**	–	**β-Hairpin**	**β-Sheet**	**Enzymes**	**β-Hairpin**
1cma-a	1azp-	1zaa-	1a31-	1ecr-	1bnz-	1ckt-	1ramA	1apl-		1bdt-	1d3u-	1bss-	1ihf-a
**Enzymes**	1bf4-		1a35-	1xbr-a	**β-Sheet**		1vkx-	1lli-		**Enzymes**	1tgh-	1ipp-	**β-Sheet**
7ice-	**Enzymes**		1bhm-a	**Enzymes**	1c9bB		**Zipper type**	**Others**		1cyq-	**Enzymes**	**Helix turn helix**	1vol-
**Helix turn helix**	2dnj-		1dnk-	1bnk-	1cdw-		1an4-	1a3qA		1dctA	2bdp-	1tc3-	**Enzymes**
3orc-	2rve-		1t7pA	1bpx-	**Enzymes**		1hlo-a	1bf5-*		1rv5-	3ktq-		1a74-a
**Other α-helices**			3bam-	1qss-	10mh-			1nfkA		4skn-	**Helix turn helix**		1ssp-
1skn-			**Helix turn helix**	1qsy-	1clq-			**Zinc coordinating group**		5mht-	1fjl-a		1vas-
**Zipper type**			6pax-	2bpf-	1pvi-a			1a1g-		**Helix turn helix**	**Zinc coordinating group**		3pvi-
1ysa-a			**Other α-helices**	2ktq-	1tau-			1aay-*		1gdt-a	1cit-		**Helix turn helix**
			1b3t-a	2ssp-	2pvi-			1d66-*		1ignA^a^			1fok-
			**Zinc coordinating group**	4ktq-	**Other α-helices**			1ubd-*		1rpe-			1hcr-a
			1lat-	**Helix turn helix**	1qrv-			1zme-		6cro-			1mnm-a
				1akh-				**Zipper type**		**Zinc coordinating group**			1yrn-a
				1hddC^a^				1an2-		2gli-a			3cro-a
				1pdn-						**Zipper type**			**Zinc coordinating group**
				3hddA						1a02-			1a6y-
				**Other α-helices**						1a0a-			
				1aoi-									
				**Zinc coordinating group**									
				1glu-									
				1tsr-a									
				2nll-									

a These protein–DNA complexes are also present in DS3 (see Materials and Methods section).

The classification was done in the SMEC region (for DS2), which implies that we have considered only highly significant interactions of the amino acid side chains with the chemical components of the DNA.

It should be noted that our classification scheme based on the interaction patterns of amino acids with nucleotide components in PDG does not directly deal with the type of interaction involved (like electrostatics, van der Waals, H-bonding, etc). However, indirectly, the P-p cluster is dominated by electrostatic interaction and the P-S clusters are composed of van der Waals interactions along with stacking of aromatic residues with the deoxyribose ring. The P-B graphs are dominated by stacking of amino acids (mostly the planar side chain of Arg) with the bases, H-bonding and also charge mediated interactions.

A comparison of the present classification with the structural motif based classification by Luscombe et al [25] shows distinct differences. A major difference is that the proteins from the same group (motif based classification) fall under different classes of interface clusters. In other words, even though the proteins have the same secondary structure motif (e.g., HTH motif), their mode of interaction may vary significantly depending on factors like the sequence of DNA (cognate/non-cognate DNA binding) and the component (p, S, or B) of the nucleotide to which it binds. However, we see a few salient features, which are common to both the classification schemes. For example, most members of the Zinc Coordinating group belong to non-overlapping category of class 6 (P-p and P-B), and non-overlapping class 4 (P-p and P-S). A few exceptions are ZIF-268 DNA complex (1zaa), Glucocorticoid mutant–DNA complex (1lat), and Reverba Orphan Nuclear Receptor/DNA complex (1a6y). Similarly, in the Helix Turn Helix members (with the exception of Matα2 Homeodomain Operator Complex (1apl), lambda repressor mutant protein/ DNA complex (1lli), and Engineered Cro monomer/DNA complex (3orc)) belong to either class 4 or class 7, both of which contain P-p and P-S clusters (overlapping as well as non-overlapping). The β-sheet group, which bind to the TATA box, belong to the classes 5 and 7, both of which contain P-S and P-B clusters. These protein–DNA complexes (with the exception of TFIIB/TBP/TATA element ternary complex (1vol)) belong to the overlapping type, indicating the continuous involvement of the deoxyribose and the base moieties of the nucleotides. As we had seen earlier, the P-S clusters in these complexes are extensive in most of the cases. Finally, a majority of the enzymes belong to the classes 4 and 7 (both overlapping and non-overlapping), which have P-p and P-S clusters (Table 4).

Here we have shown that the DBPs classification based on the protein–DNA interface interaction at the molecular level differs significantly from the protein motif based classification, although a little consensus is observed. DBPs have also been classified based on other criteria. For instance, Prabakaran et al [3] have classified DBPs based on the structural descriptors (Structural Descriptor Based Classification-SDBC), involving both protein and DNA. And Siggers et al [7] have developed a score (IAS) based on the interface geometry to align interfaces, which has been used to classify protein–DNA complexes. We have also investigated the interface clusters of the complexes from the dataset used by Prabakaran et al and found only a marginal correspondence in classification. For example, Class 1 of SDBC has prominent overlapping P-p and P-B clusters when subjected to our method of classification. This class is characterized by major groove binding proteins and interact mostly with the bases of the DNA. Also Class 2 of SDBC, which has high number of both major and minor groove contacts, and structurally deformed DNA, has overlapping P-p and P-S clusters. Class 5 of SDBC shows mostly overlapping P-S and P-B clusters.

The fact that there is only a marginal overlap between different classification schemas underscores the versatilities in protein–DNA recognition mechanism. It may be valuable to use different approaches to obtain complementary information to understand the protein–DNA recognition mechanisms in detail.

### Conclusions

The present study aims to represent a protein–DNA interface as an undirected bipartite graph based on non-covalent interactions. A quantitative method has been developed to represent the interactions between both DNA and protein as a single, combined graph. Such a representation has facilitated the study of the spatial relationships between the amino acids and the nucleotides at the protein–DNA interface in a holistic way. Thus, protein–DNA interfaces across the spectrum of complexes could be compared at a uniform level, irrespective of the structural and functional differences.

In general, we have provided a method of quantifying the interactions of proteins with the components of nucleotides (phosphate, deoxyribose and base). It is now clear that the combined representation of protein and DNA as PDGs could highlight the intricacies involved in protein–DNA recognition of some families of proteins. For instance, the predominance of protein-deoxyribose (P-S) clusters and hubs has brought out the specificity of the interaction in β-sheet proteins. Such analysis and the group specific features of protein–DNA recognition could be used as a starting point in predicting the DNA binding sites on these proteins. We have also proposed a scheme for classifying the structures based on the nature of the network connectivity present at the protein–DNA interface. Based on comparative analysis, we conclude that different classification schemes could provide complementary information on the nature of protein–DNA interactions.

Thus, the analyses performed on a dataset of protein–DNA complexes have highlighted the nature of the clusters and hubs present at the recognition site. These clusters and hubs may not only prove to be valuable in understanding the residues contributing to the stability of the protein–DNA interfaces, but also could be identified as features characteristic for a given group of proteins. The knowledge gained from the study could also provide a platform for further docking and prediction experiments.

## Materials and Methods

### Datasets

The protein–DNA complexes with resolution better than 2.5 Å and with protein identity less than 25% were taken from PDB (Version 3.1) [39] and were further cured so that the proteins (size>40 amino acids) are bound to at least one complete turn of double-stranded DNA. This resulted in a dataset (DS1) of 118 protein–DNA complexes (Table S5), which was used for evaluating the amino acid propensities for various component graphs and for recalculating the normalization values. The interface clusters and hubs were identified on two datasets, DS2 from Luscombe et al [25] and DS3 from Prabakaran et al [3], which were used for direct comparison of their classification schemes with our graph based classification scheme. DS2 was further cured for removing identical protein chains and the complexes containing single stranded DNA (Table S1).

### Construction of the Protein–DNA Graphs (PDGs)

The interaction between the amino acids and the nucleotides at the protein–DNA interface is represented as undirected bipartite Protein–DNA Graphs (PDGs). Here, the amino acids comprise one node set and the nucleotides constitute the other node set of the bipartite-PDGs as shown in Figure 1. As the focus of this work is on the protein–DNA interface, we have adopted this bipartite graph, in which the edges are made only across the amino acids and the nucleotides. The non-covalent interactions between the amino acid side chains and the nucleotides form the basis for linking the nodes. These non-covalent interactions are evaluated from the atomic contacts of the nodes. Any two atoms from nodes *i* and *j*, are considered to make a contact if they are within a distance of 4.5 Å and the total number of such contacts (*n_ij_*) is evaluated between a pair of nodes i and j. The strength of interaction (*I_ij_*) between these nodes *i* and *j* is evaluated in a manner similar to that adopted in the case of protein structure graphs [4],[23],[40], as given in Equation 1.
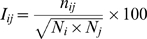
(1)where *I_ij_* is the strength of interaction between the amino acid side chain (*i*) and the nucleotide (*j*) of the protein–DNA complex. As the graphs are undirected *I_ij_* = *I_ji_*. *n_ij_* is the number of interactions existing between nodes *i* and *j* (contacts within distance of 4.5 Å). *N_i_* and *N_j_* are the normalization values of the corresponding nodes evaluated as described in the next subsection.

Here the evaluation of the strength of interaction is restricted only to atom-atom contact and does not explicitly take into account the details such as hydrogen bond, salt bridge interactions etc. Indirectly, this amounts to a measure of packing density at the selected region.

*I_ij_* is evaluated for all the amino acid-nucleotide (interface) interactions of a given protein–DNA complex. A threshold of *I_ij_* is used to connect two nodes in a graph. The threshold *I_ij_* representing the minimal atomic connectivity between interacting amino acids and the nucleotides is called as the Minimal Effective Connection (MEC). Thus, an edge between nodes i, j is defined if the *I_ij_* evaluated is greater than the user-defined MEC. For instance, a MEC of 6% specified by the user results in the generation of a PDG with nodes connected by the interaction strength *I_ij_*≥6%. Here the evaluation of *I_ij_* requires the normalization values (maximum number of contacts made by the unit) for corresponding nucleotide and amino acid units. The details of the evaluation of the normalization values are discussed below.

### Evaluation of the Normalization Values for Amino Acids (*N*_a_) and Nucleotides (*N*_n_)

The normalization values are the estimates of the maximum non-covalent contacts an amino acid or a nucleotide can have in protein–DNA complexes. The method of obtaining normalization values for amino acids in proteins was previously given [40] and a similar method is used to obtain protein–DNA normalization values. These values are evaluated from a non-redundant dataset of protein–DNA complexes [41], for all the 20 amino acids (*N*_a_) and the A, G, T, C nucleotides (*N*_n_) as shown below,
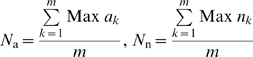
(2)where Max *a_k_* is the maximum number of non-covalent contacts made by the amino acid, “*a*,” with other amino acids and nucleotides in the protein–DNA complex “*k*.” Similarly, Max *n_k_* is evaluated for the nucleotide “*n*” in the structure “*k*.” “*m*” is the total number of protein–DNA complexes from the non-redundant dataset.

Here it should be noted that, while calculating the amino acid - amino acid contacts, the contacts made by an amino acid side chain with its sequence neighbors (*i*±1) are ignored. However, in the case of nucleotides, the sequential base contacts (stacking) of a nucleotide (*i*±1) are taken into account, ignoring only the covalent phosphate and the sugar contacts of the sequential residues. We wish to point out that the normalization values of amino acids obtained here from protein–DNA complexes are not significantly different from those obtained only from protein structures [40].

### Normalization Values of the Dissected Nucleotides

Protein-nucleic acid recognition mechanisms are often mediated by amino acids through a specific or nonspecific recognition of a nucleotide backbone or base at the protein–DNA interface. Quite often the electrostatic interactions of proteins with the phosphate groups are considered as non-specific and the stacking interactions and hydrogen bonding with bases are considered as specific. Furthermore, there is substantial conformational flexibility in the phosphodiester bond and in the conformation of the deoxyribose ring. Therefore, the nucleotides have been dissected into their chemical components such as the phosphate backbone (p), deoxyribose sugar (S), and the base (B). The interactions of an amino acid with all these individual components are characterized by constructing separate interaction graphs of amino acids with all the p, S, and B components of the DNA.

The normalization values (*N*_P_, *N*_S_, and *N*_B_) for these dissected components of the nucleotide are also calculated using Equation 3 where, in place of the whole nucleotide, the dissected component of the nucleotide is considered as,
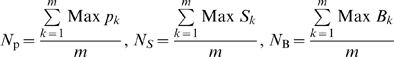
(3)where Max *p_k_* is the maximum number of non-covalent contacts, made by the phosphate with other amino acid and nucleotide residues in a protein–DNA complex “*k*.” Similarly, Max *S_k_* and Max *B_k_* are evaluated for the deoxyribose sugar and the base, respectively, from the complex “k.” “*m*” is the total number of protein–DNA complexes from the non-redundant dataset.

These individual phosphate, sugar backbone, and base-specific normalization values are useful to obtain finer details regarding the molecular connectivity existing at the protein–DNA interface. The normalization values of the individual chemical components of the nucleotide thus obtained are given in Table 5.

**Table 5:** Normalization values for the PDGs.

Nucleotides/nucleotide components	Normalization values
Phosphate (PO_4_)	25
Deoxyribose sugar	28
Adenine	144
Guanine	156
Cytosine	114
Thymine	120

The normalization values were calculated on a dataset provided by Jones et al [41]. Re-evaluation using a new updated dataset (DS1) resulted in similar normalization values (Table S5).

### Identification of Clusters of Interacting Amino Acids and Nucleotides at the Protein–DNA Interface

The PDGs are constructed as specified above and represented as binary adjacency matrices at a given MEC. Clusters of interacting nodes are identified from the adjacency matrix using Depth First Search (DFS) algorithm [42]. This is a method of graph traversal in which we obtain all the nodes that are either directly or indirectly linked to a node *V_x_* from which the search starts. The backtracking of *V_x_* starts only when all such connected nodes are explored. The next start point *V_y_* is the next unvisited node in the graph. Thus, a cluster of strongly interacting nodes (higher MEC) captures the significant interactions that exist between the amino acids and the nucleotides at the protein–DNA interface. In this study, we have focused on the interaction of protein with the components of nucleotides. Thus, we have identified the component clusters, P-p, P-S, and P-B, to capture the interaction of amino acids with the phosphate, deoxyribose and the base of the nucleotides, respectively. In many cases, amino acids interact with more than one component of a nucleotide (for example, an amino acid may interact with the phosphate atoms as well as the deoxyribose of the nucleotide). In such cases, we have defined the clusters as “overlapping clusters” (which consists of both phosphate and deoxyribose of the same nucleotide). Thus overlapping clusters constitute the interactions of amino acids captured with two or more components of a nucleotide. The details of the interface clusters for DS2 are given in Table S1 and Table S2.

### Hubs

Hubs are highly interacting nodes in a graph. In protein structure graphs, a node was declared as a hub if it was connected to a minimum of four nodes [23]. The same definition is being used here for the protein-sugar (P-S) and protein-base (P-B) hubs in PDGs. In the case of a protein-phosphate (P-p) graph, a phosphate residue connected to a minimum of three residues is considered as a hub. The purpose of hub analysis in this study is to identify the nucleic acid component, highly connected to the amino acid residues and vice versa. Thus, a (P-B) hub for example, may constitute an amino acid connected to four or more bases or a base being connected to four or more amino acid residues. The interface component hubs for the protein–DNA complexes of DS2 are presented in Table S1 and Table S3.

## Supporting Information

Figure S1 Average of the Largest Clusters (as a function of MEC) of all protein-DNA complexes in the dataset. (P-p graph in blue, P-S graph in green and P-B graph in brown). From the above plot we can see that the sizes of the largest clusters are large at lower MEC (1%–3%) and this region is classified as WMEC. There is a transition in the sizes between MEC 4%–5% (corresponding to OMEC). Beyond this transition zone, the cluster sizes decrease consistently with MEC (SMEC region). Hence we have chosen these values of MEC as cut-offs for the weak, optimal and strong MEC according to the behavior as described above, to analyze different P-p and P-B graphs. Further fine tuning was carried out based on the analysis of specific cases. In this process, we slightly modified the criteria for P-S clusters in which the OMEC was shifted to 4% to 8%. Therefore this plot gives an idea on the basis of binning the MEC (Table 1) for further analysis of the component graphs.
(0.15 MB DOC)

Figure S2 A bipartite cluster based classification of DNA-binding proteins. P-p clusters are highlighted with yellow color for phosphate and brown for interacting amino acids. P-S clusters are highlighted with green color for deoxyribose sugar and purple color for the interacting amino acids and P-B clusters are highlighted with red color for bases and blue color for the interacting amino acid residues.
(4.11 MB EPS)

Table S1 A comprehensive list of the protein-DNA complexes studied and the general clusters and hubs information.
(0.10 MB DOC)

Table S2 Component clusters in different DNA-binding proteins.
(0.18 MB DOC)

Table S3 Component hubs in DNA-binding proteins.
(0.85 MB DOC)

Table S4 Equivalent clusters in 1aoi (without linker) and 1s32 (with linker).
(0.04 MB DOC)

Table S5 Normalization values evaluated from the updated dataset (DS1).
(0.03 MB DOC)
