# Supplementary figures and images for: Insights into Protein–DNA Interactions through Structure Network Analysis
Source: PLoS Comput Biol. 2008 Sep 5;4(9):e1000170. doi: 10.1371/journal.pcbi.1000170 (PMC2518215; doi:10.1371/journal.pcbi.1000170)

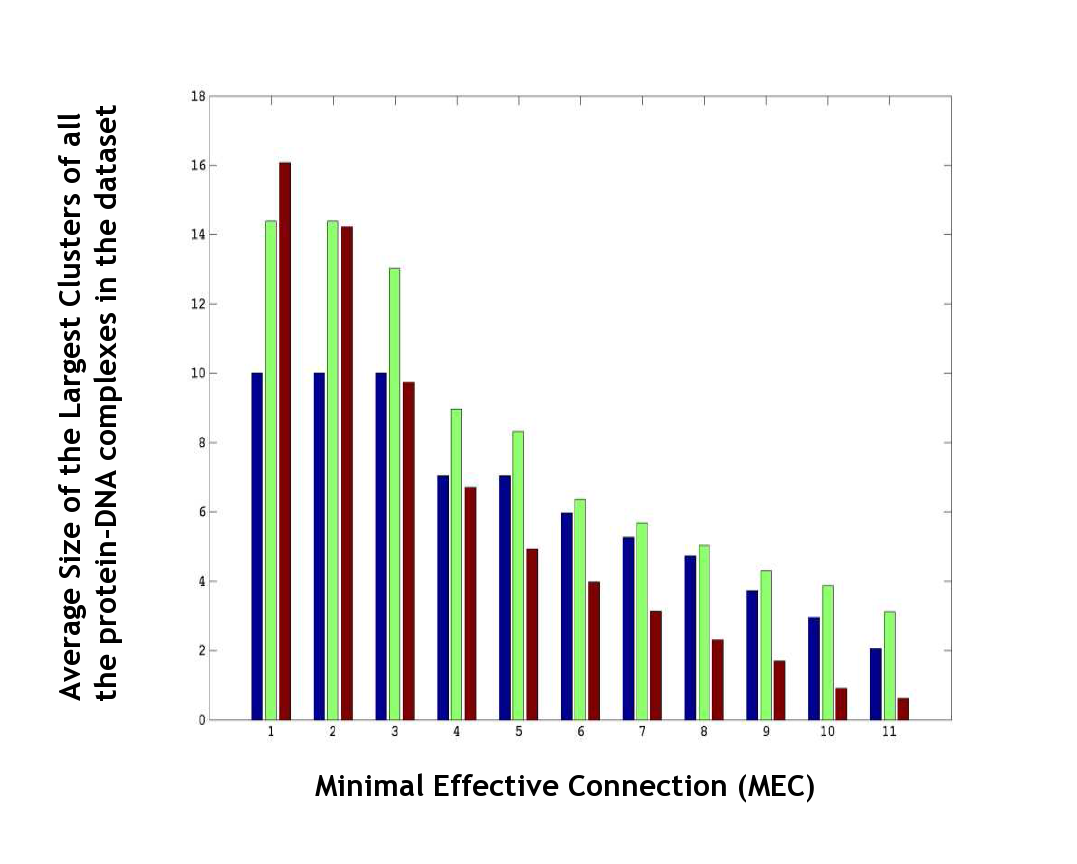

Supplement: Figure S1 — Average of the Largest Clusters (as a function of MEC) of all protein-DNA complexes in the dataset. (P-p graph in blue, P-S graph in green and P-B graph in brown). From the above plot we can see that the sizes of the largest clusters are large at lower MEC (1%–3%) and this region is classified as WMEC. There is a transition in the sizes between MEC 4%–5% (corresponding to OMEC). Beyond this transition zone, the cluster sizes decrease consistently with MEC (SMEC region). Hence we have chosen these values of MEC as cut-offs for the weak, optimal and strong MEC according to the behavior as described above, to analyze different P-p and P-B graphs. Further fine tuning was carried out based on the analysis of specific cases. In this process, we slightly modified the criteria for P-S clusters in which the OMEC was shifted to 4% to 8%. Therefore this plot gives an idea on the basis of binning the MEC (Table 1) for further analysis of the component graphs. (0.15 MB DOC) [file pcbi.1000170.s001.doc]
