# Supplementary material for: Insights into Protein–DNA Interactions through Structure Network Analysis
Source: PLoS Comput Biol. 2008 Sep 5;4(9):e1000170. doi: 10.1371/journal.pcbi.1000170 (PMC2518215; doi:10.1371/journal.pcbi.1000170)
Supplement: Table S1 — A comprehensive list of the protein-DNA complexes studied and the general clusters and hubs information. (0.10 MB DOC) [file pcbi.1000170.s003.doc]

**Table S1:** A comprehensive list of the protein-DNA complexes studied and the general clusters and hubs information.

| **GROUPS** | **PDBS** | **Number of Clusters (Avg Size of clusters)** | | | **Number of Hubs (Avg Number of Connections)** | | |
| --- | --- | --- | --- | --- | --- | --- | --- |
|  |  | **P-p** | **P-S** | **P-B** | **P-p** | **P-S** | **P-B** |
| Beta Sheet (6) | 1c9b  1cdw  1d3u  1tgh  1vol  1ytf | 2 (4.0)  1 (4.0)  3 (6.7)  3 (4.3)  4 (5.5)  2 (4.0) | 5 (6.2)  6 (6.7)  4 (8.0)  6 (6.5)  6 (5.7)  5 (6.0) | 3 (4.7)  3 (4.3)  5 (5.0)  5 (5.2)  4 (5.5)  4 (4.5) | 0(0.0)  0(0.0)  6(3.7)  1(3.0)  4(3.8)  0(0.0) | 4(4.0)  4(4.2)  3(4.0)  3(4.0)  4(4.2)  3(4.3) | 0(0.0)  0(0.0)  1(4.0)  1(4.0)  1(4.0)  0(0.0) |
| Beta Hairpin (8) | 1azp  1bdt  1bf4  1bnz  1cma  1ecr  1ihf  1xbr | 0 (0.0)  4 (8.5)  0 (0.0)  0 (0.0)  3 (5.7)  6 (6.3)  5 (6.8)  6 (6.5) | 2 (7.0)  4 (4.0)  2 (5.0)  2 (7.5)  0 (0.0)  4 (5.5)  4 (6.2)  6 (5.8) | 1 (8.0)  4 (8.2)  1 (9.0)  1 (9.0)  1 (5.0)  4 (4.5)  4 (5.2)  1 (4.0) | 0(0.0)  12(3.8)  1(3.0)  0(0.0)  4(3.8)  13(3.6)  8(3.5)  8(3.4) | 1(4.0)  0(0.0)  0(0.0)  0(0.0)  0(0.0)  1(4.0)  1(5.0)  3(4.0) | 0(0.0)  2(4.0)  0(0.0)  0(0.0)  0(0.0)  0(0.0)  0(0.0)  0(0.0) |
| Helix Turn Helix (23) | 1akh  1apl  1au7  1b72  1d3u  1fjl  1fok  1gdt  1hcr  1hdd  1ign  1lli  1mnm  1pdn  1rpe  1tc3  1vol  1yrn  3cro  3hdd  3orc  6cro  6pax | 4 (5.8)  4 (5.5)  7 (6.3)  3 (5.7)  3 (6.7)  6 (7.0)  2 (10.5)  3 (8.7)  2 (4.0)  1 (5.0)  5 (7.6)  5 (6.0)  10 (5.0)  2 (11.0)  3 (9.0)  2 (7.5)  4 (5.5)  4 (6.0)  5 (5.6)  1 (5.0)  1 (5.0)  3 (6.7)  5 (8.0) | 2 (4.5)  0 (0.0)  2 (7.0)  2 (6.0)  4 (8.0)  6 (6.2)  3 (6.0)  5 (6.8)  4 (6.5)  2 (4.5)  3 (6.7)  0 (0.0)  6 (5.8)  4 (5.8)  1 (10.0)  1 (11.0)  6 (5.7)  2 (6.5)  2 (6.0)  1 (4.0)  1 (4.0)  1 (5.0)  5 (5.8) | 3 (4.3)  1 (4.0)  4 (6.2)  2 (6.0)  5 (5.0)  3 (8.3)  3 (7.7)  6 (4.3)  1 (6.0)  1 (4.0)  4 (5.5)  3 (4.0)  6 (5.3)  1 (5.0)  2 (4.0)  2 (8.5)  4 (5.5)  3 (4.7)  2 (9.0)  1 (6.0)  0 (0.0)  3 (6.7)  1 (4.0) | 7(3.4)  5(3.2)  13(3.6)  7(3.3)  6(3.7)  21(3.5)  8(4.5)  7(3.9)  3(4.0)  3(3.3)  10(3.8)  8(3.4)  15(3.1)  4(4.2)  9(3.6)  4(3.2)  4(3.8)  6(3.3)  9(3.4)  2(3.5)  1(3.0)  11(3.5)  7(4.4) | 1(4.0)  0(0.0)  3(4.0)  0(0.0)  3(4.0)  3(4.0)  2(4.5)  4(4.2)  1(4.0)  0(0.0)  2(4.0)  0(0.0)  4(4.0)  0(0.0)  1(4.0)  2(5.5)  4(4.2)  2(4.0)  0(0.0)  0(0.0)  1(4.0)  2(4.0)  2(4.0) | 0(0.0)  2(4.0)  1(4.0)  0(0.0)  1(4.0)  2(5.0)  2(4.5)  2(4.0)  1(4.0)  0(0.0)  0(0.0)  2(4.0)  3(4.0)  0(0.0)  0(0.0)  3(4.3)  1(4.0)  2(4.0)  2(4.5)  1(5.0)  0(0.0)  4(4.5)  0(0.0) |
| Zipper Type (6) | 1a02  1a0a  1an2  1an4  1hlo  1ysa | 3 (5.7)  3 (4.0)  4 (4.5)  2 (6.0)  4 (5.5)  2 (4.5) | 1 (10.0)  1 (4.0)  2 (4.0)  1 (4.0)  0 (0.0)  0 (0.0) | 3 (5.7)  2 (5.5)  2 (5.0)  1 (7.0)  2 (8.5)  2 (6.0) | 6(3.3)  4(3.0)  2(3.0)  3(4.0)  3(3.0)  3(3.0) | 0(0.0)  0(0.0)  0(0.0)  0(0.0)  0(0.0)  0(0.0) | 1(5.0)  0(0.0)  0(0.0)  0(0.0)  0(0.0)  2(4.5) |
| Other Alpha Helices (6) | 1aoi  1b3t  1ckt  1mnm  1qrv  1skn | 16 (6.9)  6 (6.5)  0 (0.0)  10 (5.0)  0 (0.0)  2 (6.0) | 18 (4.7)  5 (6.4)  2 (7.0)  6 (5.8)  2 (7.0)  0 (0.0) | 1 (4.0)  1 (6.0)  1 (5.0)  6 (5.3)  2 (7.5)  1 (6.0) | 39(3.4)  12(4.2)  0(0.0)  15(3.1)  0(0.0)  3(3.7) | 3(4.0)  5(4.2)  1(4.0)  4(4.0)  1(5.0)  0(0.0) | 0(0.0)  0(0.0)  1(4.0)  3(4.0)  1(6.0)  1(4.0) |
| Zinc Coordinating Group (13) | 1a1g  1a6y  1aay  1cit  1d66  1glu  1lat  1tsr  1ubd  1zaa  1zme  2gli  2nll | 2 (5.5)  6 (4.8)  1 (6.0)  3 (5.7)  3 (4.3)  4 (4.8)  4 (5.2)  1 (5.0)  2 (4.0)  2 (5.0)  3 (4.0)  5 (6.0)  6 (4.8) | 0 (0.0)  4 (4.8)  0 (0.0)  2 (5.0)  0 (0.0)  2 (5.0)  2 (5.0)  1 (6.0)  0 (0.0)  0 (0.0)  1 (6.0)  1 (4.0)  1 (5.0) | 1 (14.0)  2 (5.0)  1 (16.0)  1 (7.0)  2 (5.0)  0 (0.0)  0 (0.0)  2 (4.5)  3 (6.0)  2 (10.0)  2 (5.5)  3 (5.0)  1 (5.0) | 3(3.7)  8(4.1)  4(3.0)  5(3.4)  4(3.5)  6(3.7)  7(3.6)  3(3.3)  3(3.3)  4(3.0)  6(3.0)  6(3.3)  7(3.9) | 0(0.0)  1(4.0)  0(0.0)  1(4.0)  0(0.0)  0(0.0)  0(0.0)  0(0.0)  0(0.0)  0(0.0)  1(4.0)  0(0.0)  1(4.0) | 0(0.0)  0(0.0)  0(0.0)  2(4.0)  2(4.0)  0(0.0)  0(0.0)  1(4.0)  0(0.0)  1(4.0)  2(4.5)  1(4.0)  0(0.0) |
| Enzymes (42) | 10mh  1a31  1a35  1a73  1a74  1bhm  1bnk  1bpx  1bss  1clq  1cw0  1cyq  1dct  1dnk  1ipp  1mht  1pvi  1qss  1qsy  1rv5  1ssp  1t7p  1tau  1vas  1zqa  2bdp  2bpf  2dnj  2ktq  2pvi  2rve  2ssp  3bam  3ktq  3mht  3pvi  4bdp  4ktq  4mht  4skn  5mht  7ice | 3 (4.0)  5 (6.8)  5 (8.0)  2 (11.0)  3 (5.3)  5 (5.4)  2 (7.0)  3 (5.3)  6 (4.5)  0 (0.0)  3 (5.7)  2 (6.0)  2 (5.5)  2 (4.5)  2 (11.0)  4 (5.5)  3 (4.7)  2 (8.5)  2 (7.0)  6 (5.0)  1 (5.0)  3 (5.7)  3 (4.3)  3 (5.0)  1 (4.0)  2 (5.0)  3 (4.3)  0 (0.0)  2 (7.0)  4 (4.0)  0 (0.0)  1 (4.0)  8 (5.5)  2 (9.5)  4 (5.5)  7 (5.4)  4 (5.8)  1 (9.0)  4 (4.5)  2 (5.5)  4 (4.2)  2 (4.5) | 1 (10.0)  5 (4.8)  4 (8.0)  4 (5.0)  2 (8.0)  1 (11.0)  1 (7.0)  3 (5.0)  3 (6.0)  2 (7.0)  3 (7.7)  2 (9.0)  3 (5.0)  2 (7.0)  3 (6.3)  1 (11.0)  3 (4.0)  3 (6.0)  3 (6.7)  2 (4.0)  1 (5.0)  1 (6.0)  3 (4.7)  2 (5.0)  0 (0.0)  6 (5.0)  1 (5.0)  1 (8.0)  2 (4.5)  2 (4.0)  2 (7.0)  1 (8.0)  3 (7.0)  4 (6.2)  2 (8.0)  3 (4.0)  3 (7.3)  3 (4.7)  1 (11.0)  2 (4.5)  1 (5.0)  0 (0.0) | 1 (8.0)  0 (0.0)  0 (0.0)  3 (6.7)  3 (7.3)  3 (6.0)  1 (4.0)  1 (5.0)  2 (14.0)  1 (8.0)  2 (10.5)  2 (6.0)  3 (6.7)  0 (0.0)  5 (6.0)  1 (8.0)  2 (12.5)  1 (4.0)  1 (6.0)  2 (11.5)  1 (4.0)  0 (0.0)  2 (4.5)  2 (5.5)  0 (0.0)  1 (8.0)  1 (4.0)  0 (0.0)  1 (5.0)  1 (26.0)  0 (0.0)  1 (4.0)  4 (5.2)  2 (5.0)  2 (8.5)  2 (10.5)  1 (12.0)  0 (0.0)  1 (8.0)  1 (5.0)  2 (6.5)  0 (0.0) | 5(3.4)  8(4.2)  10(4.4)  8(3.8)  6(3.7)  8(4.6)  3(3.7)  4(5.5)  14(3.5)  1(4.0)  6(3.7)  4(4.0)  3(3.0)  3(4.0)  7(3.9)  5(3.4)  9(3.6)  5(3.6)  7(3.6)  9(3.7)  2(4.0)  9(3.1)  3(3.0)  6(3.3)  2(4.0)  6(3.2)  4(3.8)  2(3.0)  6(3.5)  8(3.8)  2(5.0)  1(6.0)  10(5.1)  6(3.7)  6(3.3)  10(4.1)  8(3.2)  4(3.5)  6(3.3)  1(5.0)  5(3.6)  3(3.7) | 0(0.0)  2(4.5)  3(4.3)  4(4.0)  1(4.0)  1(4.0)  2(4.0)  2(4.0)  2(4.0)  4(4.5)  3(4.0)  2(4.0)  2(5.0)  4(4.5)  3(4.7)  0(0.0)  2(5.0)  2(4.5)  1(4.0)  1(5.0)  0(0.0)  1(4.0)  1(4.0)  0(0.0)  2(4.0)  0(0.0)  1(7.0)  1(4.0)  0(0.0)  0(0.0)  1(5.0)  2(4.0)  2(5.0)  3(5.0)  0(0.0)  4(4.2)  0(0.0)  3(5.0)  1(5.0)  2(4.0)  0(0.0) | 0(0.0)  0(0.0)  0(0.0)  0(0.0)  0(0.0)  2(4.0)  0(0.0)  0(0.0)  4(4.0)  1(4.0)  1(4.0)  0(0.0)  0(0.0)  0(0.0)  1(4.0)  0(0.0)  0(0.0)  0(0.0)  1(4.0)  2(4.0)  1(4.0)  0(0.0)  1(4.0)  1(5.0)  0(0.0)  1(4.0)  0(0.0)  0(0.0)  0(0.0)  2(4.0)  0(0.0)  0(0.0)  4(4.2)  0(0.0)  2(5.0)  0(0.0)  1(4.0)  0(0.0)  0(0.0)  0(0.0)  0(0.0)  0(0.0) |
| Others (6) | 1a02  1a3q  1bf5  1nfk  1ram  1vkx | 3 (5.7)  3 (5.3)  2 (6.5)  2 (7.5)  3 (6.0)  4 (7.0) | 1 (10.0)  1 (5.0)  0 (0.0)  1 (4.0)  0 (0.0)  2 (4.0) | 3 (5.7)  1 (9.0)  2 (4.5)  3 (4.0)  2 (5.5)  4 (5.8) | 6(3.3)  3(3.3)  2(5.5)  4(3.2)  3(3.3)  7(3.7) | 0(0.0)  0(0.0)  0(0.0)  0(0.0)  0(0.0)  0(0.0) | 1(5.0)  1(4.0)  0(0.0)  0(0.0)  0(0.0)  1(4.0) |

(Clusters: MEC_(P-p)_=4%, MEC_(P-S)_=5%, MEC_(P-B)_=3%)

(Hubs: MEC_(P-p)_=3%, MEC_(P-S)_=4%, MEC_(P-B)_=3%)

In column 1, the number of protein-DNA complexes is given brackets.
