# Supplementary material for: Insights into Protein–DNA Interactions through Structure Network Analysis
Source: PLoS Comput Biol. 2008 Sep 5;4(9):e1000170. doi: 10.1371/journal.pcbi.1000170 (PMC2518215; doi:10.1371/journal.pcbi.1000170)
Supplement: Table S2 — Component clusters in different DNA-binding proteins. (0.18 MB DOC) [file pcbi.1000170.s004.doc]

**Table S2** Component clusters in different DNA Binding Proteins.

| **Group** | **Component Clusters** | **PDBs** | **Clusters*** |
| --- | --- | --- | --- |
| (b) Beta Sheet | P-p | 1c9bB  1cdw-  1d3u-  1tgh-  1vol-  1ytfA | 1) B1184LYS B1188ARG C1410T C1411T D1431G  2) B1256TYR B1265LYS B1268GLN B1283ARG B1286TYR C1402G C1403A  3) B1277VAL B1278THR B1281THR B1285ARG D1441T D1442T    1) C121T C122A A303SER A305LYS    1) D115C D116C A248ARG A249SER A252SER A280VAL A284THR    1) F7T F8A A209SER A211LYS |
|  | P-S | 1c9bB  1cdw-  1d3u-  1tgh-  1vol-  1ytfA | 1) D107T D108T D109T B167ASN B210THR B212LEU B222THR B223GLY  2) C14A C15G B214PHE B216SER B218LYS  3) C11A C12A B257ASN B313THR B314GLY  4) C10T B294ARG B301VAL B303LEU  5) D111T D112A B305PHE B309LYS    1) C108T C109T A163ASN A218THR  2) B11A B12G A210PHE A214LYS  3) B8A B9A A253ASN A309THR A310GLY  4) C111T C112A C113G A285PRO A301PHE A305LYS  5) B7T A288ILE A290ARG A297VAL A299LEU    1) A13ASN A68THR A69GLY D1435T D1436T  2) A44PRO A60PHE C1416T C1417A C1418C  3) A104ASN A160GLY C1413A C1414A  4) A135PRO A151PHE A155LYS B1245LYS D1438A D1439A D1440C    1) C117T C118A C119T A163ASN A197ILE A206THR A208LEU A218THR A219GLY A221LYS  2) B103T B104A A283LEU A284PHE  3) C121T C122A C123C A285PRO A301PHE A305LYS  4) B105T B106A A288ILE A297VAL A299LEU A309THR A310GLY    1) B27ASN B70THR B82THR B85LYS D109T D110T  2) B61ILE B63ARG B72LEU D108T  3) C10A C11G B74PHE B78LYS  4) C7A C8A B117ASN B173THR B174GLY  5) C6T B152ILE B154ARG B161VAL B163LEU  6) D112T D113A B165PHE B169LYS    1) F3T F4T F5T A69ASN A112THR A114LEU A124THR  2) E15A E16C A116PHE A120LYS  3) E12A E13A A159ASN A215THR A216GLY  4) F7T F8A F9T A191PRO A207PHE A211LYS |
|  | P-B | 1c9bB  1cdw-  1d3u-  1tgh-  1vol-  1ytfA | 1) C11A C12A B169VAL B257ASN B313THR    1) B8A B9A A165VAL A253ASN A309THR  2) B6A C112A A284PHE A285PRO    1) A13ASN A106VAL D1435T D1436T  2) A15VAL A104ASN C1413A C1414A  3) A43PHE A44PRO C1417A D1433A    1) C118A C119T A163ASN A255VAL  2) B110A C115T C116A C117T A193PHE A208LEU  3) B108A B109T A210PHE A216VAL  4) B106A B107T A253ASN A309THR  5) B103T B104A C122A A284PHE A285PRO    1) B27ASN D109T D110T B119VAL  2) C6T C7A C8A B29VAL B117ASN B173THR  3) C10A C11G B57PHE B74PHE D107T  4) C4T C5A D113A B148PHE B149PRO    1) E12A E13A A71VAL A159ASN A215THR  2) F1G F2T E16C A99PHE  3) F8A E9T E10A A190PHE A191PRO |
| (b) Beta Hairpin | P-p | 1azp-  1bdt-  1bf4-  1bnz-  1cma-  1ecr-  1ihf-  1xbr- | 1) E3T A4MET E4A B10PHE A32SER A33VAL  2) A10PHE F14C F15T F16C B23ARG B32SER B33VAL B34ASN  3) F3T C4MET F4G C32SER C33VAL  4) D4MET C10PHE E14T E15T E16C D23ARG D32SER D33VAL D34ASN        1) D12G A40ARG A52THR A53ASN A54SER  2) C4G B40ARG B52THR B53ASN B54SER    1) A91ALA A93ARG A157ARG C334T C335T  2) A129THR A139ARG A156TYR B321A  3) A174ASN A285VAL A287HIS B314T  4) A178ILE A180ASN A229LYS B317C B318A    1) A4THR A5LYS B46ARG E46C E47A  2) C-32A C-31G C-30C E36C A55ARG A57LYS A78VAL A80THR A82ARG A88LYS    1) A67ARG A196TYR A205LYS D517G  2) A99ARG A101LYS C502A C503T  3) B64LYS B148ASN B149LYS D505T D506C  4) B67ARG B196TYR B205LYS C517G  5) B68ARG B145LYS B209ASN C515G C516T  6) B99ARG B101LYS D502A D503T |
|  | P-S | 1azp-  1bdt-  1bf4-  1bnz-  1cma-  1ecr-  1ihf-  1xbr- | 1) A7LYS A8TYR A9LYS A44ALA C113T C114C  2) A24TRP A26VAL B103G B104A    1) E2A E3T A4MET A32SER  2) F2A F3T C4MET C32SER  3) D4MET E13C E14T D32SER    1) A8TYR A9LYS A45ALA C113A C114C  2) A22LYS A24TRP A26VAL B103G B104T    1) A8TYR A9LYS A29MET A45ALA A47SER C77A C78T C79T  2) A22LYS A24TRP B69A B70T      1) A89LYS A235LYS B322T C333G    1) C-43A C-42C A4THR B44GLU E45G B46ARG  2) E35T E36C E37A E38A A60ARG A64ASN A71ILE A73ILE A76ARG    1) A67ARG A68ARG A209ASN A210PRO A211PHE C512T D515G D516T  2) A196TYR A206ILE A215PHE D517G D518T  3) A213LYS A214ALA C510C C511C  4) B67ARG B68ARG B209ASN B210PRO B211PHE D512T C515G C516T  5) B206ILE B215PHE C517G C518T  6) B213LYS B214ALA D510C D511C |
|  | P-B | 1azp-  1bdt-  1bf4-  1bnz-  1cma-  1ecr-  1ihf-  1xbr- | 1) A24TRP A26VAL B102C B103G    1) E3T A4MET E4A B13ARG  2) E6T E7A A9GLN B9GLN A11ASN B11ASN A13ARG F15T F16C F17T F18A  3) F3T C4MET F4G D13ARG  4) F6T F7A C9GLN D9GLN C11ASN D11ASN C13ARG E15T E16C E17T E18A    1) A24TRP A26VAL B102C B103G    1) A24TRP A26VAL A29MET B68A B69A C78T C79T      1) A241ARG A250GLN A288ARG B312A    1) C-38T E37A E38A A65PRO A66LYS  2) C-43A E44T E45G B46ARG  3) C-29A C-28A D29T B59ARG B62ARG B63ASN B64PRO    1) B67ARG B215PHE C516T C517G |
| (c) Helix Turn Helix | P-p | 1akh-  1apl-  1au7-  1b72-  1d3u-  1fjl-  1fok-  1gdt-  1hcr-  1hddC  1ignA  1lli-  1mnm-  1pdn-  1rpe-  1tc3-  1vol-  1yrn-  3cro-  3hddA  3orc-  6cro-  6pax- | 1) C15T C16A A119ILE A122ARG  2) C37T C38A B136PHE B175GLN B179TRP  3) C3C C4A B156TYR B177LYS B184ARG    1) A15T A16A C136PHE C175GLN  2) A2C A3A D156TYR D184ARG    1) A20ARG A26THR A27GLN A48CYS A52ASN C459T C460A  2) A42PHE A43SER A46THR A59ASN A62LYS D483T D484G  3) A146ARG A153ARG C451C C452T  4) B17LYS B20ARG B26THR B27GLN B48CYS B52ASN D487T D488A  5) B42PHE B56SER B59ASN C454A C455T  6) B103LYS B106THR C464A C465T    1) E25C E26C A227TYR A252GLN A255ARG  2) D9A B240PHE B279GLN B282ASN  3) E29T E30C B260TYR B266LYS B288ARG    1) B1184LYS B1188ARG C1410T C1411T D1431G  2) B1256TYR B1265LYS B1268GLN B1283ARG B1286TYR C1402G C1403A  3) B1277VAL B1278THR B1281THR B1285ARG D1441T D1442T    1) E7C E8T E9G A25TYR A53ARG A57ARG  2) B3ARG E6T A31ARG A46GLN  3) D8A D9G B25TYR B53ARG B57ARG    1) A112ARG A298THR A299ASN C935C  2) A218ASN A422ARG C938C C939C    1) F26T F27C A145LYS A146ILE A148ARG A171ALA A174THR A177LYS  2) D26T D27C B145LYS B146ILE B148ARG B170ILE B174THR    1) B8T A142ARG A178ARG A179TYR    1) C25TYR B27G B28T C53ARG    1) D30G D31G A383THR A444SER A445ILE A448LYS  2) C7A C8C A399THR A401ASN A591ASN A593ASN  3) C12C C13A A449PHE A544ARG A548PHE A575LYS A578THR    1) D3T D4A A19LYS A22TYR A33GLN A52ASN  2) E14G E15T A42MET A50LEU A61ASN  3) E3T E4A E5T B19LYS B22TYR B26LYS B33GLN B52ASN  4) D14G D15T B42MET B61ASN    1) E2A A29LYS A30THR A33HIS  2) A35THR A39ARG B46LYS F46A F47G  3) E13G E14G B35THR B39ARG A46LYS  4) F36T B37SER B40LYS B70TYR C134HIS  5) E21T E22A C130PRO C135ARG C136PHE C175GLN  6) F28C F29G C156TYR C184ARG  7) E7C E8T D132ARG D134HIS  8) F50A F51A D136PHE D175GLN D179TRP    1) C6ASN C7GLN B26T B27G B28A C46SER  2) C23ARG B24C B25G C49CYS C52LYS C56ARG    1) L16ASN L17GLN A23A A24A A25A L36ASN  2) B13C L42PRO L43ARG L44PHE  3) A33C A34A R39THR R40LYS R44PHE    1) A5G A6G A7G A8G C224SER C225LEU C226HIS C230ARG C240ARG  2) B109A B110G C234ARG C235SER C238CYS C249TYR    1) D115C D116C A248ARG A249SER A252SER A280VAL A284THR    1) C14T C15T C16A A100LYS A115ARG A122ARG  2) C37T C38A B136PHE B175GLN B179TRP  3) C3C C4A B156TYR B184ARG    1) B2A B3T L10ARG L16THR L17GLN  2) A15T A16T L27LYS L30SER  3) A14C L39THR L42PRO L44PHE  4) A4T A5A R10ARG R16THR R17GLN R32GLN    1) A25TYR A53ARG D327G D328T    1) S6A S7T A25VAL A26TYR    1) R3T U3T R4A U4A A15GLY A16GLN A31ASN A35HIS  2) S15T T15T S16G T16G A25VAL A26TYR  3) S13G T13G S14G T14G A38ARG A56LYS    1) A6ASN A7GLN A12PHE A46SER C2018T C2019G C2020A  2) A18LEU A23ARG A49CYS A52LYS A56ARG C2016C C2017G  3) A35ARG A36PRO A37CYS B1006T B1007T  4) A75VAL A76ALA A116SER A119SER A122ARG B1020T B1021G  5) A95PHE A96ALA A97TRP A121ASN A125ARG C2003C C2004T C2005G |
|  | P-S | 1akh-  1apl-  1au7-  1b72-  1d3u-  1fjl-  1fok-  1gdt-  1hcr-  1hddC  1ignA  1lli-  1mnm-  1pdn-  1rpe-  1tc3-  1vol-  1yrn-  3cro-  3hddA  3orc-  6cro-  6pax- | 1) C8A C9A C38A C39C B132ARG  2) C11A C12A C36T B135ARG      1) A27GLN A103LYS A105ARG C457T C458A C459T D491C  2) B27GLN B105ARG C463C D486A D487T    1) D11T D12G A207ARG A208THR A210PHE  2) D8G E37A B237ARG B239ASN B240PHE    1) A13ASN A68THR A69GLY D1435T D1436T  2) A44PRO A60PHE C1416T C1417A C1418C  3) A104ASN A160GLY C1413A C1414A  4) A135PRO A151PHE A155LYS B1245LYS D1438A D1439A D1440C    1) A2ARG D5A D6T E13A  2) F1T D4A A5ARG A8PHE E14T  3) B2ARG B3ARG E5A E6T D12T D13A B44ARG  4) E3T E4A B5ARG B8PHE  5) C2ARG C3ARG F5A F6T  6) D1A F3T F4A C5ARG C6THR C8PHE    1) A95GLN A102ILE A106SER A110PHE B904G    1) C13A C14A F25A F26T A142ARG A144ARG A145LYS A146ILE A174THR  2) E13A E14A D25A D26T B142ARG B144ARG B145LYS B146ILE B174THR    1) B8T C27A A140ARG A143ALA A175THR  2) B15A C19T A177TYR A185ILE  3) B12A B13A B14G A187LYS A188ARG A189MET      1) C5A C6C D37C A360LYS A363PHE A399THR A402SER  2) C13A C14C D29T A384THR A445ILE A446LYS A449PHE A537THR      1) E6C E7C E8T E9A A16LYS A17GLU A19ARG D131TYR D132ARG D134HIS  2) E12A F45T A46LYS B46LYS  3) E20T F36T F37T C135ARG    1) A13G A14A C69GLY C70GLY    1) B12T B13C A32A A33C L39THR R39THR L43ARG    1) B108T B109A C202PRO C204GLY C238CYS  2) A12T A13A B110G C203ARG    1) B27ASN B70THR B82THR B85LYS D109T D110T  2) B61ILE B63ARG B72LEU D108T  3) C10A C11G B74PHE B78LYS  4) C7A C8A B117ASN B173THR B174GLY  5) C6T B152ILE B154ARG B161VAL B163LEU  6) D112T D113A B165PHE B169LYS    1) C8A C9A C38A C39C B132ARG  2) C11T C12T C36T B135ARG    1) B11A A14C L30SER L43ARG L44PHE  2) A12T B12G R39THR R43ARG    1) A5ARG A8PHE C211T C212A      1) S15T T15T S16G T16G A26TYR    1) A17PRO A66ARG A68ILE C2015G C2016C  2) A69GLY A70GLY B1017G B1018A  3) A71SER A74ARG A75VAL B1019G B1020T |
|  | P-B | 1akh-  1apl-  1au7-  1b72-  1d3u-  1fjl-  1fok-  1gdt-  1hcr-  1hddC  1ignA  1lli-  1mnm-  1pdn-  1rpe-  1tc3-  1vol-  1yrn-  3cro-  3hddA  3orc-  6cro-  6pax- | 1) B26T B27G B28T C185ARG    1) A44GLN A48CYS C459T C460A  2) A49ARG B154GLN D481C D482A D483T D484G  3) A105ARG A151ASN C458A D490A D491C D492A  4) B49ARG A154GLN C453C C454A C455T C456G    1) D11T D12G D13A A207ARG A253ASN  2) D7T D8G E36T B237ARG B290ARG    1) A13ASN A106VAL D1435T D1436T  2) A15VAL A104ASN C1413A C1414A  3) A43PHE A44PRO C1417A D1433A    1) A2ARG D2A D3T D4A A5ARG D5A E14T A51ASN  2) B2ARG E2G E3T E4A B5ARG E5A D12T D14C B51ASN  3) C2ARG F2G F3T F4A C5ARG F5A C51ASN    1) A12GLN A222SER C936A C937T  2) A13ASN A225LYS B904G B905A  3) A79ARG A105TRP B906T B907G C934T C935C  4) A95GLN A217ASN A228ARG B901T B902C B903G C939C C940G    1) C16T E21A E22A A130ARG  2) C5T C6G C7T A172ARG A176TYR  3) E5T E6G E7T B172ARG B176TYR    1) B10A B11T C23C A174SER A190ASN      1) D28G D29T D30G D31G D32G A385HIS A404ARG A408ARG A589PRO  2) C7A C8C A401ASN A591ASN  3) D22T D23G D24G A542ARG A546ARG    1) A4LYS E13A E14G A55ASN  2) B4LYS D13G D14G B55ASN    1) E8T E9A A19ARG A38LYS F47G  2) E14G E15G B34VAL F37T B38LYS F38T3) E20T F35A F36T B37SER C135ARG  4) E21T E22A E23C F33A C132ARG C178ASN C182ASN  5) F30T F31G F32T C185ARG  6) E1G E2A E3T D185ARG      1) A24A A25A L28GLN L32GLU  2) B3T B4A R28GLN R32GLU    1) A10C A12T A13A A15A B107A B108T B109A B110G B111G C202PRO C203ARG C237HIS  2) A6G A7G A8G C226HIS C236ARG    1) B27ASN D109T D110T B119VAL  2) C6T C7A C8A B29VAL B117ASN B173THR  3) C10A C11G B57PHE B74PHE D107T  4) C4T C5A D113A B148PHE B149PRO    1) C24T C25G C26A A120ASN A124ARG  2) C8A C37T C38A B132ARG B182ASN  3) C5T C6G C7T B185ARG    1) B3T B4A B5C A16T L28GLN L29GLN L32GLN  2) A4T A5A R28GLN R32GLN  3) A6C B15T B16G B17T R29GLN        1) S15T T15T S16G T16G A26TYR A28SER  2) R3T U3T R4A U4A A27GLN  3) S13G T13G S14G T14G A32LYS |
| (d) Zipper Type | P-p | 1a02-  1a0a-  1an2-  1an4-  1hlo-  1ysa- | 1) N424TYR N426THR N520LYS N522ARG N523ASN B5017T B5018T  2) N538LYS N572ARG N665ARG B5015T    1) C4A C5C A12ARG B41SER  2) D6C D7C B12ARG B15ARG    1) B11C B12G A33ARG A36ARG  2) D9C D10A A35ARG C60ARG  3) B9C B10A C35ARG A60ARG  4) D11C D12G C33ARG C36ARG    1) A211ARG B240LYS C308T C309C  2) B210ARG B211ARG A239SER A240LYS D329C D330C    1) A23ARG A26ARG C107C C108G  2) B23ARG B26ARG D120C D121G |
|  | P-S | 1a02-  1a0a-  1an2-  1an4-  1hlo-  1ysa- | 1) N537ARG N538LYS N572ARG N665ARG A4011T A4012G B5014A B5015T    1) C4A A12ARG B39ALA B41SER |
|  | P-B | 1a02-  1a0a-  1an2-  1an4-  1hlo-  1ysa- | 1) N421ARG N430ARG N571GLN A4003G A4004G A4005A  2) N424TYR N427GLU B5017T B5018T B5019C  3) F147ASN A4015T A4016C B5005A B5006T    1) C5C C6A A9GLU D12T  2) B2ARG B5HIS C9T C10G C11G    1) D9C B13T A28HIS A29ASN A32GLU  2) B9C D13T C28HIS C29ASN C32GLU    1) A208GLU A211ARG C308T C309C D334T    1) A18HIS A19ASN A22GLU A25ARG C109T C110G D118C  2) B19ASN B22GLU B25ARG C105C D122T    1) A11T A12C B28A C235ASN |
| (e) Other Alpha Helices | P-p | 1aoi-  1b3t-  1ckt-  1mnm-  1qrv-  1skn- | 1) A41TYR A46VAL A47ALA J229A J230C  2) A42ARG A43PRO A45THR I68G I144G I145A  3) B32PRO B36ARG I60C A63ARG A64LYS J238T  4) A65LEU A66PRO A69ARG J237T  5) C29ARG I30A C32ARG D36ILE J268G  6) C75LYS C76THR J278C J279A  7) E42ARG E43PRO E45THR J215C J290G  8) F32PRO F36ARG E63ARG E64LYS I91T J207A  9) F79LYS F80THR I100G I101C  10) G75LYS G76THR I132C I133A  11) H83ARG H84SER H85THR J186G J187A    1) C112G C113C C114T A467LYS A472GLY A518TYR A521ARG A522ARG A536LEU B554LEU  2) D202G A516SER A519ASN A590THR  3) D212G D213C D214T B467LYS B518TYR B521ARG B522ARG B536LEU A554LEU  4) C102G B516SER B519ASN B590THR      1) E2A A29LYS A30THR A33HIS  2) A35THR A39ARG B46LYS F46A F47G  3) E13G E14G B35THR B39ARG A46LYS  4) F36T B37SER B40LYS B70TYR C134HIS  5) E21T E22A C130PRO C135ARG C136PHE C175GLN  6) F28C F29G C156TYR C184ARG  7) E7C E8T D132ARG D134HIS  8) F50A F51A D136PHE D175GLN D179TRP      1) A9T A10C P460LYS P507ARG P508ARG  2) B3G B4G B5G P482LEU P503ARG P506ARG P510LYS |
|  | P-S | 1aoi-  1b3t-  1ckt-  1mnm-  1qrv-  1skn- | 1) A40ARG A46VAL J229A J230C  2) A41TYR A49ARG I143T J152T J153A  3) A43PRO I67A I68G J228A  4) B32PRO I60C A63ARG A65LEU A66PRO J237T  5) I50C B79LYS A83ARG J246G  6) I39G C42ARG D84SER J257A  7) I5A I6T I7A E39HIS E41TYR E49ARG  8) E40ARG E46VAL I82A I83A  9) E43PRO I81G J214G J215C  10) F30THR F32PRO E63ARG E65LEU E66PRO I90T J207A  11) E83ARG I99A J196C J197A  12) G42ARG H84SER I111A I112T J185G  13) G76THR G77ARG I131G J165A    1) D206G D207C D208A A462GLY A463GLY A464TRP  2) C112G D210A A469ARG A518TYR B538ARG  3) C110A D212G B469ARG B518TYR A538ARG  4) C106G C107C C108A B462GLY B463GLY B464TRP      1) E6C E7C E8T E9A A16LYS A17GLU A19ARG D131TYR D132ARG D134HIS  2) E12A F45T A46LYS B46LYS  3) E20T F36T F37T C135ARG    1) A7ARG C9G  2) C5T C6A A12TYR A40GLY A43TRP |
|  | P-B | 1aoi-  1b3t-  1ckt-  1mnm-  1qrv-  1skn- | 1) A37PHE B108G B109G C208C C209C    1) E8T E9A A19ARG A38LYS F47G  2) E14G E15G B34VAL F37T B38LYS F38T  3) E20T F35A F36T B37SER C135ARG  4) E21T E22A E23C F33A C132ARG C178ASN C182ASN  5) F30T F31G F32T C185ARG  6) E1G E2A E3T D185ARG    1) C3G C4A C5T C6A A12TYR A13MET D16A A33THR A36ALA    1) B7T A10C P507ARG P511ASN |
| (f) Zinc Coordinating Group | P-p | 1a1g-  1a6y-  1aay-  1cit-  1d66-  1glu-  1lat-  1tsr-  1ubd-  1zaa-  1zme-  2gli-  2nll- | 1) B7G A116PHE A125HIS A128ILE    1) A124PHE A127ARG A154GLN D630T  2) A150ARG A151ASN A157ARG D631G  3) B124PHE B127ARG B154GLN D622T  4) B150ARG B151ASN B157ARG D623G    1) B7G A114ARG A116PHE A125HIS    1) A244TYR A301VAL A303ARG B408A B409G  2) A255PHE A258ARG A285GLN C423T  3) A281ARG A282ASN A288ARG C424G    1) A20LYS A23LYS E35C E36C  2) D13C D14C B44THR B46ARG A51ARG  3) B9GLN B10ALA B15ARG D15T    1) C3T A463PHE A474TYR A493PRO  2) C4G A489ARG A490LYS A496ARG  3) D3T B459SER B489ARG B496ARG    1) D10A D11T A463PHE A472HIS A473ASN A493PRO  2) D12G A489ARG A490LYS A496ARG  3) C12G B489ARG B490LYS B496ARG    1) E13G B239ASN B241SER B275CYS    1) B30A B31T C343HIS C346VAL      1) B15C B16C C31SER C43LYS    1) D64A D65A A151PHE A160HIS A163ARG A183ARG  2) D58C D59C A209LYS A220HIS A224THR  3) D56G D57A A229LYS A242TYR    1) A158PHE A161ARG A188GLN D532T  2) A184ARG A185ASN A191ARG D533G  3) B315TYR B328ARG B376LEU C514G  4) B326PHE B329ARG B358GLN D522T  5) B332GLN B381ARG B385ARG C515G  6) B354ARG B355ASN B361ARG D523G |
|  | P-S | 1a1g-  1a6y-  1aay-  1cit-  1d66-  1glu-  1lat-  1tsr-  1ubd-  1zaa-  1zme-  2gli-  2nll- | 1) A113TYR A171VAL A173PHE C605A D638T  2) B113TYR A151ASN B171VAL B173PHE C613A D630T      1) A309GLY A311ARG B409G B410G C428T C429T  2) A313ARG A316SER C431T C432C      1) D-9C D-8A A452TYR A511LYS    1) D4C D5A B451HIS B452TYR B510ARG    1) E12T F14T B241SER B248ARG            1) B332GLN B381ARG B384LYS C514G |
|  | P-B | 1aoi-  1b3t-  1ckt-  1mnm-  1qrv-  1skn- | 1) A37PHE B108G B109G C208C C209C    1) E8T E9A A19ARG A38LYS F47G  2) E14G E15G B34VAL F37T B38LYS F38T  3) E20T F35A F36T B37SER C135ARG  4) E21T E22A E23C F33A C132ARG C178ASN C182ASN  5) F30T F31G F32T C185ARG  6) E1G E2A E3T D185ARG    1) C3G C4A C5T C6A A12TYR A13MET D16A A33THR A36ALA    1) B7T A10C P507ARG P511ASN |

*the nodes in the clusters are given as <chain id><residue id><residue name>

(MEC _(P-p)_ = 7%, MEC _(P-S)_ = 8%, MEC _(P-B)_ = 4%)

(Enzymes and Others not reported)
