# Supplementary material for: Insights into Protein–DNA Interactions through Structure Network Analysis
Source: PLoS Comput Biol. 2008 Sep 5;4(9):e1000170. doi: 10.1371/journal.pcbi.1000170 (PMC2518215; doi:10.1371/journal.pcbi.1000170)
Supplement: Table S3 — Component hubs in DNA-binding proteins. (0.85 MB DOC) [file pcbi.1000170.s005.doc]

**Table S3** Component Hubs in DNA Binding Proteins

| **PDBS** | **P-p** | **P-S** | **P-B** |
| --- | --- | --- | --- |
| **Beta Sheet**  1c9bB |  | C10T | B197PHE* |
|  |  | C11A | B288PHE* |
|  |  | D107T |  |
|  |  | D108T |  |
|  |  |  |  |
|  |  |  |  |
| 1cdw- |  | B7T | A284PHE* |
|  |  | B8A |  |
|  |  | C107T |  |
|  |  | C108T |  |
|  |  |  |  |
|  |  |  |  |
| 1d3u- | C1402G | C1412T | A43PHE |
|  | C1403A | C1413A |  |
|  | C1411T | D1435T |  |
|  | D1440C |  |  |
|  | D1441T |  |  |
|  | D1442T |  |  |
|  |  |  |  |
| 1tgh- | C117T | B105T | A193PHE |
|  |  | B106A |  |
|  |  | C118A |  |
|  |  |  |  |
| 1vol- | C6T | C6T | B57PHE |
|  | D108T | C7A |  |
|  | D115C | D108T |  |
|  | D116C | D109T |  |
|  |  |  |  |
| 1ytfA |  | E11T |  |
|  |  | E12A |  |
|  |  | F4T |  |
| **Beta Ribbons**  1azp |  | C114C |  |
|  |  |  |  |
| 1bdt | C4MET |  | A11ASN |
|  | E2A |  | C11ASN |
|  | E3T |  |  |
|  | E4A |  |  |
|  | E13C |  |  |
|  | E14T |  |  |
|  | E15T |  |  |
|  | F2A |  |  |
|  | F3T |  |  |
|  | F4G |  |  |
|  | F14C |  |  |
|  | F15T |  |  |
|  |  |  |  |
| 1bf4 | C116C |  |  |
|  |  |  |  |
| 1bnz |  |  |  |
|  |  |  |  |
| 1cma | C2T |  |  |
|  | C4G |  |  |
|  | D11A |  |  |
|  | D12G |  |  |
|  | D1442T |  |  |
|  |  |  |  |
| 1ecr | A178ILE | B320C |  |
|  | B312A |  |  |
|  | B314T |  |  |
|  | B315T |  |  |
|  | B316A |  |  |
|  | B317C |  |  |
|  | B318A |  |  |
|  | B321A |  |  |
|  | B322T |  |  |
|  | C330T |  |  |
|  | C334T |  |  |
|  | C335T |  |  |
|  | C336G |  |  |
|  |  |  |  |
| 1ihf | C-42C | B46ARG |  |
|  | C-41A |  |  |
|  | C-31G |  |  |
|  | C-29A |  |  |
|  | C-20T |  |  |
|  | D24A |  |  |
|  | E36C |  |  |
|  | E46C |  |  |
|  |  |  |  |
| 1xbr | C503T | C516T |  |
|  | C505T | C517G |  |
|  | C516T | D517G |  |
|  | C517G |  |  |
|  | D503T |  |  |
|  | D505T |  |  |
|  | D516T |  |  |
|  | D517G |  |  |
| **Helix Turn Helix**  1akh- | C3C | B132ARG |  |
|  | C4A |  |  |
|  | C15T |  |  |
|  | C25G |  |  |
|  | C26A |  |  |
|  | C37T |  |  |
|  | C38A |  |  |
|  |  |  |  |
| 1au7- | C451C | A105ARG | A105ARG |
|  | C454A | B105ARG |  |
|  | C456G | C459T |  |
|  | C459T |  |  |
|  | C460A |  |  |
|  | C463C |  |  |
|  | C464A |  |  |
|  | D479C |  |  |
|  | D482A |  |  |
|  | D483T |  |  |
|  | D484G |  |  |
|  | D487T |  |  |
|  | D488A |  |  |
|  |  |  |  |
| 1b72- | D8G |  |  |
|  | D9A |  |  |
|  | D12G |  |  |
|  | D13A |  |  |
|  | E25C |  |  |
|  | E26C |  |  |
|  | E30C |  |  |
|  |  |  |  |
| 1d3u- | C1402G | C1412T | A43PHE |
|  | C1403A | C1413A |  |
|  | C1411T | D1435T |  |
|  | D1440C |  |  |
|  | D1441T |  |  |
|  | D1442T |  |  |
|  |  |  |  |
| 1fjl- | A25TYR | A2ARG | A5ARG |
|  | A53ARG | A5ARG | B5ARG |
|  | A57ARG | B2ARG |  |
|  | B25TYR |  |  |
|  | B53ARG |  |  |
|  | B57ARG |  |  |
|  | D4A |  |  |
|  | D5A |  |  |
|  | D6T |  |  |
|  | D7C |  |  |
|  | D8A |  |  |
|  | D8A |  |  |
|  | E4A |  |  |
|  | E5A |  |  |
|  | E6T |  |  |
|  | E7C |  |  |
|  | E8T |  |  |
|  | E8T |  |  |
|  | F4A |  |  |
|  | F5A |  |  |
|  | F6T |  |  |
|  |  |  |  |
| 1fok- | B902C | B904G | A105TRP |
|  | B904G | C934T | A217ASN |
|  | B905A |  |  |
|  | C934T |  |  |
|  | C935C |  |  |
|  | C936A |  |  |
|  | C937T |  |  |
|  | C938C |  |  |
|  |  |  |  |
| 1gdt- | C5T | A142ARG | A142ARG |
|  | D26T | B142ARG | B173SER |
|  | D27C | D26T |  |
|  | E6G | F26T |  |
|  | E15T |  |  |
|  | F26T |  |  |
|  | F27C |  |  |
|  |  |  |  |
| 1hcr- | B8T | B8T | A190ASN |
|  | B9G |  |  |
|  | C20T |  |  |
|  |  |  |  |
| 1hddC | A13A |  |  |
|  | B27G |  |  |
|  | B28T |  |  |
|  |  |  |  |
| 1ignA | C5A | A360LYS |  |
|  | C6C | A446LYS |  |
|  | C7A |  |  |
|  | C12C |  |  |
|  | C13A |  |  |
|  | C14C |  |  |
|  | D21C |  |  |
|  | D22T |  |  |
|  | D29T |  |  |
|  | D30G |  |  |
|  |  |  |  |
| 1lli- | D3T |  | A45SER |
|  | D4A |  | B45SER |
|  | D15T |  |  |
|  | E3T |  |  |
|  | E4A |  |  |
|  | E13A |  |  |
|  | E14G |  |  |
|  | E15T |  |  |
|  |  |  |  |
|  |  |  |  |
| 1mnm- | E2A | A19ARG | A19ARG |
|  | E10A | C132ARG | B38LYS |
|  | E13G | E1G | C135ARG |
|  | E14G | F35A |  |
|  | E21T |  |  |
|  | E22A |  |  |
|  | F28C |  |  |
|  | F29G |  |  |
|  | F35A |  |  |
|  | F36T |  |  |
|  | F44T |  |  |
|  | F45T |  |  |
|  | F46A |  |  |
|  | F47G |  |  |
|  | F50A |  |  |
|  |  |  |  |
| 1pdn- | A3C |  |  |
|  | B24C |  |  |
|  | B25G |  |  |
|  | B26T |  |  |
|  |  |  |  |
| 1rpe- | B3T | L43ARG |  |
|  | B4A |  |  |
|  | B12T |  |  |
|  | B13C |  |  |
|  | B14T |  |  |
|  | A24A |  |  |
|  | A25A |  |  |
|  | A33C |  |  |
|  | A34A |  |  |
|  |  |  |  |
| 1tc3- | A6G | B109A | C202PRO |
|  | A7G | C203ARG | C203ARG |
|  | A8G |  | C237HIS |
|  | B110G |  |  |
|  |  |  |  |
| 1vol- | C6T | C6T | B57PHE |
|  | D108T | C7A |  |
|  | D115C | D108T |  |
|  | D116C | D109T |  |
|  |  |  |  |
| 1yrn- | C3C | B132ARG | A124ARG |
|  | C15T | B135ARG | B185ARG |
|  | C25G |  |  |
|  | C26A |  |  |
|  | C37T |  |  |
|  | C38A |  |  |
|  |  |  |  |
| 3cro- | A4T |  | L29GLN |
|  | A13T |  | R29GLN |
|  | A14C |  |  |
|  | A15T |  |  |
|  | B3T |  |  |
|  | B4A |  |  |
|  | B12G |  |  |
|  | B13T |  |  |
|  | B14T |  |  |
|  |  |  |  |
| 3hddA | C212A |  | A5ARG |
|  | D327G |  |  |
|  |  |  |  |
| 3orc- | S6A | S5G |  |
|  |  |  |  |
| 6cro- | A15GLY | A26TYR | A27GLN |
|  | A16GLN | A31ASN | A28SER |
|  | A17THR |  | A31ASN |
|  | A26TYR |  | A32LYS |
|  | A38ARG |  |  |
|  | R3T |  |  |
|  | R4A |  |  |
|  | S15T |  |  |
|  | T15T |  |  |
|  | U3T |  |  |
|  | U4A |  |  |
|  |  |  |  |
| 6pax- | B1007T | B1019G |  |
|  | B1020T | B1020T |  |
|  | B1021G |  |  |
|  | C2004T |  |  |
|  | C2016C |  |  |
|  | C2017G |  |  |
|  | C2018T |  |  |
| **Zipper Type**  1a02- | A4010T |  | F147ASN |
|  | A4011T |  |  |
|  | B5015T |  |  |
|  | B5016T |  |  |
|  | B5017T |  |  |
|  | B5018T |  |  |
|  |  |  |  |
| 1a0a- | C4A |  |  |
|  | C5C |  |  |
|  | D7C |  |  |
|  | D8C |  |  |
|  |  |  |  |
| 1an2- | B12G |  |  |
|  | D12G |  |  |
|  |  |  |  |
| 1an4- | C309C |  |  |
|  | D329C |  |  |
|  | D330C |  |  |
|  |  |  |  |
| 1hlo- | C104C |  |  |
|  | C105C |  |  |
|  | D118C |  |  |
|  |  |  |  |
| 1ysa- | A6A |  | C235ASN |
|  | A7T |  | D235ASN |
|  | B28A |  |  |
| **Other Alpha Helices**  1aoi- | A42ARG | E41TYR |  |
|  | A63ARG | E83ARG |  |
|  | E42ARG | G42ARG |  |
|  | E63ARG |  |  |
|  | H27ARG |  |  |
|  | I30A |  |  |
|  | I31G |  |  |
|  | I32T |  |  |
|  | I40G |  |  |
|  | I50C |  |  |
|  | I51A |  |  |
|  | I60C |  |  |
|  | I61A |  |  |
|  | I70T |  |  |
|  | I71G |  |  |
|  | I81G |  |  |
|  | I82A |  |  |
|  | I90T |  |  |
|  | I91T |  |  |
|  | I101C |  |  |
|  | I112T |  |  |
|  | I122G |  |  |
|  | I132C |  |  |
|  | I144G |  |  |
|  | J177G |  |  |
|  | J178T |  |  |
|  | J186G |  |  |
|  | J207A |  |  |
|  | J217G |  |  |
|  | J218A |  |  |
|  | J228A |  |  |
|  | J229A |  |  |
|  | J230C |  |  |
|  | J237T |  |  |
|  | J238T |  |  |
|  | J247C |  |  |
|  | J268G |  |  |
|  | J278C |  |  |
|  | J290G |  |  |
|  |  |  |  |
| 1b3t- | C102G | C101G |  |
|  | C103G | C102G |  |
|  | C111T | D201G |  |
|  | C112G | D202G |  |
|  | C113C | D213C |  |
|  | C114T |  |  |
|  | D202G |  |  |
|  | D203G |  |  |
|  | D211T |  |  |
|  | D212G |  |  |
|  | D213C |  |  |
|  | D214T |  |  |
|  |  |  |  |
| 1ckt- |  | C207T | A37PHE |
|  |  |  |  |
| 1mnm- | E2A | A19ARG | A19ARG |
|  | E10A | C132ARG | B38LYS |
|  | E13G | E1G | C135ARG |
|  | E14G | F35A |  |
|  | E21T |  |  |
|  | E22A |  |  |
|  | F28C |  |  |
|  | F29G |  |  |
|  | F35A |  |  |
|  | F36T |  |  |
|  | F44T |  |  |
|  | F45T |  |  |
|  | F46A |  |  |
|  | F47G |  |  |
|  | F50A |  |  |
|  |  |  |  |
| 1nfkA | C5A |  |  |
|  | C6A |  |  |
|  | C8T |  |  |
|  | C9C |  |  |
|  |  |  |  |
| 1qrv- |  | C5T | A13MET |
|  |  |  |  |
| 1skn- | A9T |  | P511ASN |
|  | A10C |  |  |
|  | B4G |  |  |
| **Zinc Coordinating Group**  1a1g- | B6G |  |  |
|  | B7G |  |  |
|  | B8G |  |  |
|  |  |  |  |
| 1a6y- | C605A | D631G |  |
|  | C606G |  |  |
|  | C613A |  |  |
|  | C614G |  |  |
|  | D622T |  |  |
|  | D623G |  |  |
|  | D630T |  |  |
|  | D631G |  |  |
|  |  |  |  |
| 1aay- | B5T |  |  |
|  | B6G |  |  |
|  | B7G |  |  |
|  | B8G |  |  |
|  |  |  |  |
| 1cit- | B408A | A311ARG | A312GLY |
|  | B409G |  | A313ARG |
|  | C423T |  |  |
|  | C424G |  |  |
|  | C432C |  |  |
|  |  |  |  |
| 1d66- | D14C |  | A18LYS |
|  | D15T |  | B18LYS |
|  | D16C |  |  |
|  | E35C |  |  |
|  |  |  |  |
| 1glu- | C-8A |  |  |
|  | C3T |  |  |
|  | C4G |  |  |
|  | D-9C |  |  |
|  | D-8A |  |  |
|  | D3T |  |  |
|  |  |  |  |
| 1lat- | C5A |  |  |
|  | C6G |  |  |
|  | C11T |  |  |
|  | C12G |  |  |
|  | D5A |  |  |
|  | D11T |  |  |
|  | D12G |  |  |
|  |  |  |  |
| 1tsr- | E2T |  | B280ARG |
|  | E12T |  |  |
|  | E13G |  |  |
|  |  |  |  |
| 1ubd- | B28A |  |  |
|  | B31T |  |  |
|  | B34A |  |  |
|  |  |  |  |
| 1zaa- | A4G |  | C18ARG |
|  | A5T |  |  |
|  | A7G |  |  |
|  | A8G |  |  |
|  |  |  |  |
| 1zme- | A13C | B11C | C41HIS |
|  | A14T |  | D41HIS |
|  | B11C |  |  |
|  | B12T |  |  |
|  | B14C |  |  |
|  | B15C |  |  |
|  |  |  |  |
| 2gli- | C3T |  | A246SER |
|  | D56G |  |  |
|  | D57A |  |  |
|  | D59C |  |  |
|  | D65A |  |  |
|  | D66G |  |  |
|  |  |  |  |
| 2nll- | C513A | C514G |  |
|  | C514G |  |  |
|  | C515G |  |  |
|  | D522T |  |  |
|  | D523G |  |  |
|  | D532T |  |  |
|  | D533G |  |  |
| **Enzymes**  10mh- | B403C |  |  |
|  | C425A |  |  |
|  | C426G |  |  |
|  | C428G |  |  |
|  | C429C |  |  |
|  |  |  |  |
| 1a31- | C7A |  |  |
|  | C8C |  |  |
|  | D112A |  |  |
|  | D113A |  |  |
|  | D114A |  |  |
|  | D115G |  |  |
|  | D116T |  |  |
|  | D117C |  |  |
|  |  |  |  |
| 1a35- | C7A | A532LYS |  |
|  | C8C | C11A |  |
|  | C10T |  |  |
|  | C11A |  |  |
|  | C12G |  |  |
|  | D113A |  |  |
|  | D114A |  |  |
|  | D115G |  |  |
|  | D116T |  |  |
|  | D117C |  |  |
|  |  |  |  |
| 1a73- | C3G | C14G |  |
|  | C14G | D13A |  |
|  | C15A | D14G |  |
|  | D3G |  |  |
|  | D4A |  |  |
|  | D13A |  |  |
|  | D14G |  |  |
|  | D15A |  |  |
|  |  |  |  |
| 1a74- | C403G | C413A |  |
|  | C414G | C414G |  |
|  | C415A | D413A |  |
|  | D403G | D414G |  |
|  | D414G |  |  |
|  | D415A |  |  |
|  |  |  |  |
| 1bhm- | C4G | D7T | A155ARG |
|  | C5G |  | B155ARG |
|  | C6A |  |  |
|  | C7T |  |  |
|  | D4G |  |  |
|  | D5G |  |  |
|  | D6A |  |  |
|  | D7T |  |  |
|  |  |  |  |
| 1bnk- | D8T | D8T |  |
|  | D9T |  |  |
|  | E23G |  |  |
|  |  |  |  |
| 1bpx- | T10C | D1G |  |
|  | P8C | D2T |  |
|  | D1G |  |  |
|  | D2T |  |  |
|  |  |  |  |
| 1bss- | C902A | C905A | A186THR |
|  | C903A | D805A | B186THR |
|  | C905A |  | C908T |
|  | C906T |  | D808T |
|  | C907A |  |  |
|  | C908T |  |  |
|  | C909C |  |  |
|  | D802A |  |  |
|  | D803A |  |  |
|  | D805A |  |  |
|  | D806T |  |  |
|  | D807A |  |  |
|  | D809C |  |  |
|  | D810T |  |  |
|  |  |  |  |
| 1clq- | D2C | E2G | D2C |
|  |  | D3G |  |
|  |  |  |  |
| 1cw0- | M304T | M308T | M309G |
|  | M308T | M309G |  |
|  | M309G | O354T |  |
|  | N353C | O355A |  |
|  | O355A |  |  |
|  | O356G |  |  |
|  |  |  |  |
| 1cyq- | C403G | C414G |  |
|  | C415A | D513A |  |
|  | D503G | D514G |  |
|  | D515A |  |  |
|  |  |  |  |
| 1dctA | F11C | F11C |  |
|  | F12A | N1T |  |
|  | F13C |  |  |
|  |  |  |  |
| 1dnk- | C314A | C314A |  |
|  | C315C | C315C |  |
|  | C316C |  |  |
|  |  |  |  |
| 1ipp- | C203G | C213A | A57ASN |
|  | C204A | C214G |  |
|  | C214G | D213A |  |
|  | C215A | D214G |  |
|  | D203G |  |  |
|  | D214G |  |  |
|  | D215A |  |  |
|  |  |  |  |
| 1mht- | B403A | C426G |  |
|  | C425A | C427+C |  |
|  | C426G | C428G |  |
|  | C427+C |  |  |
|  | C428G |  |  |
|  |  |  |  |
| 1pvi- | C3G |  |  |
|  | C9C |  |  |
|  | C10T |  |  |
|  | C11G |  |  |
|  | D5C |  |  |
|  | D8G |  |  |
|  | D9C |  |  |
|  | D10T |  |  |
|  | D11G |  |  |
|  |  |  |  |
| 1qrv- |  | C5T | A13MET |
|  |  |  |  |
| 1qss- | B107G | B110G |  |
|  | B108G | C204C |  |
|  | B109C |  |  |
|  | B111C |  |  |
|  | C204C |  |  |
|  |  |  |  |
| 1qsy- | B107G | B110G | C204T |
|  | B108G | C204T |  |
|  | B109C |  |  |
|  | B111C |  |  |
|  | C204T |  |  |
|  | C208G |  |  |
|  | C213G |  |  |
|  |  |  |  |
| 1rv5- | C2A | D5A | C8T |
|  | C3A |  | D8T |
|  | C5A |  |  |
|  | C6T |  |  |
|  | C9C |  |  |
|  | C10T |  |  |
|  | D2A |  |  |
|  | D6T |  |  |
|  | D9C |  |  |
|  |  |  |  |
| 1ssp- | A6A | A6A | E272LEU |
|  | A7T |  |  |
|  |  |  |  |
| 1t7pA | P17T |  |  |
|  | P18G |  |  |
|  | P21A |  |  |
|  | T4C |  |  |
|  | T7G |  |  |
|  | T9C |  |  |
|  | T10A |  |  |
|  | T11C |  |  |
|  | T13G |  |  |
|  |  |  |  |
| 1tau- | T908G | P951C | A583ASN |
|  | P955A |  |  |
|  | P951C |  |  |
|  |  |  |  |
| 1vas- | B207T | B206G | C221A |
|  | B208T |  |  |
|  | B210C |  |  |
|  | B211G |  |  |
|  | B212C |  |  |
|  | C223G |  |  |
|  |  |  |  |
| 1zqa- | T4T |  |  |
|  | P5A |  |  |
|  |  |  |  |
|  |  |  |  |
|  |  |  |  |
| 2bdp- | P13A | P14T | T26A |
|  | P15G | T27G |  |
|  | P16C |  |  |
|  | T28C |  |  |
|  | T30T |  |  |
|  | T35C |  |  |
|  |  |  |  |
| 2bpf- | T8G |  |  |
|  | P5G |  |  |
|  | P6C |  |  |
|  | P7C |  |  |
|  |  |  |  |
| 2dnj- | B306C | C314C |  |
|  | C314C |  |  |
|  |  |  |  |
| 2ktq- | B107G | D205G |  |
|  | B108G |  |  |
|  | B109C |  |  |
|  | B111C |  |  |
|  | D208G |  |  |
|  | D213G |  |  |
|  |  |  |  |
| 2pvi- | C3G |  | A84HIS |
|  | C8G |  | B84HIS |
|  | C10T |  |  |
|  | C11G |  |  |
|  | D8G |  |  |
|  | D10T |  |  |
|  | D11G |  |  |
|  | D12G |  |  |
|  |  |  |  |
| 2rve- | D8G |  |  |
|  | F8G |  |  |
|  |  |  |  |
| 2ssp- | A7T | A6A |  |
|  |  |  |  |
| 3bam- | C3T | C7T | A155ARG |
|  | C4G | E3T | A197GLY |
|  | C5G |  | A198MET |
|  | C6A |  | B155ARG |
|  | C7T |  |  |
|  | C8C |  |  |
|  | D4G |  |  |
|  | E1G |  |  |
|  | E2A |  |  |
|  | E3T |  |  |
|  |  |  |  |
| 3ktq- | B107G | B110G |  |
|  | B108G | D204G |  |
|  | B109C |  |  |
|  | B111C |  |  |
|  | D208G |  |  |
|  | D213G |  |  |
|  |  |  |  |
| 3mht- | C403A | D426G | A237GLN |
|  | D425A | D427C | D427C |
|  | D426G | D428G |  |
|  | D427C |  |  |
|  | D428G |  |  |
|  | D429C |  |  |
|  |  |  |  |
| 3pvi- | C3G |  |  |
|  | C8G |  |  |
|  | C9C |  |  |
|  | C10T |  |  |
|  | C11G |  |  |
|  | D3G |  |  |
|  | D8G |  |  |
|  | D9C |  |  |
|  | D10T |  |  |
|  | D11G |  |  |
|  |  |  |  |
| 4bdp- | P20C | P29A | T3A |
|  | P24A | T2T |  |
|  | P25T | T3A |  |
|  | P26G | T4T |  |
|  | P28A |  |  |
|  | T5T |  |  |
|  | T7C |  |  |
|  | T12T |  |  |
|  |  |  |  |
| 4ktq- | B107G |  |  |
|  | B108G |  |  |
|  | B109C |  |  |
|  | B111C |  |  |
|  |  |  |  |
| 4mht- | C403A | D426G |  |
|  | D425A | D427+C |  |
|  | D426G | D428G |  |
|  | D427+C |  |  |
|  | D428G |  |  |
|  | D429C |  |  |
|  |  |  |  |
| 4skn- | A7G | A6G |  |
|  |  |  |  |
|  |  |  |  |
| 5mht- | C403C | D427C |  |
|  | D425A | D428G |  |
|  | D426G |  |  |
|  | D427C |  |  |
|  | D428G |  |  |
|  |  |  |  |
| 7ice- | T4T |  |  |
|  | P5T |  |  |
|  | P6G |  |  |
| **Others**  1a02- | A4010T |  | F147ASN |
|  | A4011T |  |  |
|  | B5015T |  |  |
|  | B5016T |  |  |
|  | B5017T |  |  |
|  | B5018T |  |  |
|  |  |  |  |
| 1a3qA | C509A |  | A52ARG |
|  | C511T |  |  |
|  | C512C |  |  |
|  | F15T |  |  |
|  |  |  |  |
| 1bf5- | B1010C |  |  |
|  | C2006T |  |  |
|  |  |  |  |
| 1nfkA | C5A |  |  |
|  | C6A |  |  |
|  | C8T |  |  |
|  | C9C |  |  |
|  |  |  |  |
| 1ramA | C10A |  |  |
|  | C12T |  |  |
|  | C13T |  |  |
|  |  |  |  |
| 1vkx- | C2G |  | B354ARG |
|  | C6A |  |  |
|  | C7C |  |  |
|  | C9T |  |  |
|  | C10T |  |  |
|  | D17A |  |  |
|  | D19G |  |  |

(MEC _(P-p)_=3%, MEC _(P-S)_=4%, MEC _(P-B)_=3%)
