# Supplementary material for: Insights into Protein–DNA Interactions through Structure Network Analysis
Source: PLoS Comput Biol. 2008 Sep 5;4(9):e1000170. doi: 10.1371/journal.pcbi.1000170 (PMC2518215; doi:10.1371/journal.pcbi.1000170)
Supplement: Table S4 — Equivalent clusters in 1aoi (without linker) and 1s32 (with linker). (0.04 MB DOC) [file pcbi.1000170.s006.doc]

**Table S4** Equivalent clusters in 1aoi (without linker) and 1s32 (with linker).

| **P-P at MEC = 5%** | | **P-S at MEC = 5%** | |
| --- | --- | --- | --- |
| **1aoi** | **1s32** | **1aoi** | **1s32** |
| I 29 A  C 32 ARG  I 30 A  D 36 ILE  J 268 G  C 29 ARG | C 832 ARG  I 30 A  D 1236 ILE  J 268 G  C 829 ARG | D 84 SER  D 85 THR  I 39 G  C 44 GLY  J 257 A  C 42 ARG | D 1284 SER  D 1285 THR  I 39 G  C 844 GLY  J 257 A  C 842 ARG |
| H 36 ILE  I 122 G  G 32 ARG  J 176 A  G 29 ARG | H 1437 TYR  I 121 G  H 1436 ILE  I 122 G  G 1029 ARG | H 36 ILE  H 37 TYR  I 121 G  G 29 ARG | I 122 G  J 174 A  H 1430 ARG  H 1436 ILE  H 1437 TYR  I 121 G  G 1029 ARG |
| H 84 SER  H 85 THR  J 186 G  J 187 A  H 83 ARG | H 1484 SER  H 1485 THR  J 186 G  J 187 A  H 1483 ARG | I 111 A  I 112 T  H 84 SER  J 185 G  G 42 ARG | G 1044 GLY  I 111 A  H 1484 SER  J 185 G  G 1042 ARG |
|  | D 1284 SER  D 1285 THR  I 40 G  I 41 A  D 1283 ARG |  | D 1227 ARG  I 28 A  J 268 G  D 1230 ARG  I 29 A  C 832 ARG |
|  |  |  | I 103 G  I 104 T  D 1226 ARG  J 192 G  D 1224 LYS |

Only the clusters around the linker region are reported. For a given component graph the equivalent clusters (clusters that are common in both structures) occupy the same row. The new clusters due to linker binding do not have a counterpart in 1aoi. This information is a supplement to the Figure 8.
