# Supplementary material for: Insights into Protein–DNA Interactions through Structure Network Analysis
Source: PLoS Comput Biol. 2008 Sep 5;4(9):e1000170. doi: 10.1371/journal.pcbi.1000170 (PMC2518215; doi:10.1371/journal.pcbi.1000170)
Supplement: Table S5 — Normalization values evaluated from the updated dataset (DS1). (0.03 MB DOC) [file pcbi.1000170.s007.doc]

Table S5: Normalization values evaluated from the updated dataset (DS1).

| Nucleotides/ Nucleotide Components | Normalization Values |
| --- | --- |
| Phosphate (PO_4_)  Deoxyribose Sugar  Adenine  Guanine  Cytosine  Thymine | 27  31  148  158  116  116 |

The above normalization values were calculated for the dataset DS1. These values are similar to the values that are used in generating PDGs (Table 5). The pdb codes for the protein-DNA complexes in the dataset are provided in the table below [39].

List of protein-DNA complexes in DS1

| 1am9 1jj4 1r7m 2cgp 2nll 1au7 1k4t 1rh6 2d5v 2oaa 1b3t 1k78 1rio 2ddg 2ofi 1b94 1ku7 1rm1 2dnj 2opf 1bdt 1skn 2dtu 2owo 1bl0 1l3l 1ssp 2p0j 1cw0 1le8 1sxq 2etw 2pi0 1lq1 1tc3 2ex5 2pyj 1d2i 1mj2 1tkd 2q10 1d3u 1tro 2fjx 2q2t 1dc1 1mus 1u8b 2fkc 2qkb 1diz 1nfk 1vrl 2r1j 1ebm 1nh2 1w0u 2g1p 2vla 1eyu 1njz 1w7a 2gih 2yvh 1f44 1nkp 1wd0 2h27 1f4k 1nlw 2h7g 3bep 1f6o 1oe4 2h7h 3bs1 1gxp 1ozj 2hap 1h6f 1pm5 1zs4 2hos 3btx 1h89 1pp7 2ady 2hvr 3c25 1hcr 1pt3 2alz 2i06 3c2i 1hlv 1puf 2aor 2i13 3clz 1i3j 2aq4 2ihm 3cro 1iaw 2bam 2iie 6pax 1ign 2bnw 2is6 1j1v 2c6y 2isz 1je8 1qum 2c7o 2c9l 2jg3 |
| --- |
